# AI Agents as Universal Task Solvers

**DOI:** 10.3390/e28030332

**Published:** 2026-03-16

**Authors:** Alessandro Achille, Stefano Soatto

**Affiliations:** 1 AWS AI and Caltech, Amazon Agentic AI, Los Angeles, CA, USA; 2 AWS AI and UCLA, Amazon Agentic AI, Los Angeles, CA, USA

**Keywords:** generative AI, computability, algorithmic information, large language models, inductive learning, transductive inference, reasoning, scaling laws, dynamical systems, Occam’s Razor

## Abstract

We describe AI agents as stochastic dynamical systems and frame the problem of learning to reason as in *transductive inference*: Rather than approximating the distribution of past data as in classical induction, the objective is to capture its *algorithmic structure* so as to reduce the time needed to solve new tasks. In this view, information from past experience serves not only to reduce a model’s uncertainty, as in Shannon’s classical theory, but to reduce the computational effort required to find solutions to unforeseen tasks. Working in the verifiable setting, where a checker or reward function is available, we establish three main results. First, we show that the optimal speed-up for a new task is tightly related to the algorithmic information it shares with the training data, yielding a theoretical justification for the power-law scaling empirically observed in reasoning models. Second, while the *compression view* of learning, rooted in Occam’s Razor, favors simplicity, we show that transductive inference yields its greatest benefits precisely when the data-generating mechanism is most complex. Third, we identify a possible failure mode of naïve scaling: in the limit of unbounded model size and computing, models with access to a reward signal can behave as *savants*, brute-forcing solutions without acquiring transferable reasoning strategies. Accordingly, we argue that a critical quantity to optimize when scaling reasoning models is *time*, the role of which in learning has remained largely unexplored.

## 1. Introduction

Much of machine learning focuses on *inductive inference*: fitting a function to past data and expecting it to generalize to similar inputs. While valuable, this perspective is incomplete. In an agentic setting, we want a pre-trained model to *reason* at inference time about the structure of a *specific instance of a novel task*, rather than simply apply a map fixed during training. From this alternative viewpoint, the role of learning shifts from building a fixed classifier to equipping a solver with the knowledge needed to efficiently tackle previously unseen problems. This perspective draws on what Vapnik termed *transductive inference* [1]: reasoning directly about specific instances rather than applying a mapping fixed at training time.

Many tasks in machine learning can be, and historically have been, approached inductively: classifying an object, for example, relies mainly on similarity with past observations rather than reasoning about the instance itself. However, AI is increasingly tackling problems where this framework falls short. Consider writing code that passes a given set of unit tests, proving a theorem with access to a proof checker, or finding a protein configuration that minimizes an energy function. In these cases, the task is not implicitly defined by past examples, as in inductive learning, but rather by, at least approximately, a known *verifier* or *reward signal* capable of evaluating any candidate solution. More precisely, each problem instance *x* is paired with a function f(x,y) that can score or verify any candidate solution *y*.

This setting poses a fundamentally different challenge from inductive learning. Finding a correct solution is, in principle, always *achievable*: one could enumerate candidates *y* until one satisfies f(x,y). This brute-force strategy guarantees success, provided we are willing to wait a time exponential in the length of the solution. Unlike in inductive learning, the core difficulty is therefore not accuracy or generalization, which can always be ensured given enough time, but *computational efficiency*: quickly finding a solution to a previously unseen task *f* on an instance *x*.

For any fixed task, one could of course design or train a *bespoke solver*, carefully optimized for that specific problem, that finds solutions extremely fast. But could there exist a *general* solver that tackles any unseen task nearly as efficiently as the best task-specific solver?

That would seem too good to be true, and likely to violate some kind of “no free lunch theorem.” Yet, Levin [2] and Solomonoff [3] showed that a *universal solver U* can solve any instance *x* of a task *essentially* as well as a solver *A* that is optimal for that task. In particular, the universal solver finds a solution in time $T_{U}$ bounded by

$$T_{U}\left(x\right)\;\leq \;2^{\ell (A)}\;T_{A}\left(x\right),$$

where ℓ(A) is the description length of *A* and $T_{A}\left(x\right)$ is the optimal time to find a solution to that instance. Crucially, the universal solver requires only a constant factor longer than a task-specific solver, where the constant depends on the *complexity of the optimal solver*, not on the particular instance *x*. The catch is that such constant factor $2^{\ell (A)}$ can be astronomically large.

This is where *learning* comes in: In [3] Solomonoff observed that, even if a task has never been faced before, prior experience lets us encode effective problem-solving programs more succinctly (e.g., by reusing components of the solution) thereby reducing the factor $2^{\ell (A)}$. Thus, in the transductive setting with no uncertainty on the reward, the value of learning is measured *not* by a reduction in error rate as in induction but in the reduction of the *time* it takes to find solutions to unforeseen tasks. This points to a foundational principle for transductive learning: Rather than trying to capture the statistical structure (joint distribution) of past data in hope that future data will respect it, as in induction, in transduction we want to capture the shared algorithmic structure of past data, given which an agent can reason to quickly find solutions to *new* computable tasks. Accordingly, to be able to solve *general* unseen tasks, an agent should *learn to perform transductive inference; that is, they should “learn to reason”.*

Learning transductive inference as envisioned by Solomonoff in 1984 [3] has been mostly an academic concern for decades because it seems to require (meta-)learning a conditional distribution over *programs* that, when run through a universal Turing machine (UTM), solve a given task. This has been impractical until recently. But modern large-scale models, such as large language models (LLMs) or reasoning models, can effectively encode distributions over programs. Moreover, they themselves serve, loosely speaking, as a powerful new type of *computation engine*—planning, searching, calling tools, and coordinating multi-step reasoning—which, however, fundamentally differs from UTMs and does not cleanly fit either the theory of Solomonoff and Levin or the classical mold of inductive learning.

In this work, we embrace the transductive view and formalize learning in a way that fits modern reasoning models. We show in what sense an LLM, despite being stochastic and in many ways antithetical to Turing machines, is a valid model of universal computation. We then contextualize Levin’s guarantees and Solomonoff’s vision using LLMs instead of UTMs, which requires entirely different proof techniques, study resource-aware objectives, and ground these ideas into practical algorithms that trade inference-time computing to generalize to novel problems not seen during training.

Our analysis focuses on universal solvers in a verifiable setting, where an oracle is provided to compute the reward or verify a solution. This restriction yields a clean theoretical framework, but it is admittedly narrow: most real-world problems involve both an inductive component—reducing uncertainty from data—and a transductive component—reasoning efficiently about a specific instance. Classical statistical learning theory addresses the former; our theory aims to address the latter. We do not tackle the general case in which the two are intertwined, where reasoning is required and the reward, or an efficient approximation of it, must itself be learned. Nevertheless, we believe that the basic mechanisms we identify—enabling a model to become a universally efficient solver in the spirit of Solomonoff, and the tradeoffs they reveal among learning, algorithmic information, and time—extend beyond the purely verifiable setting.

### 1.1. Can an LLM-Powered AI Agent Be a Universal Solver?

Levin and Solomonoff showed that a universal solver exists, but the construction hinges on using (deterministic) universal Turing machines (UTMs). LLMs, by contrast, are neither Turing machines nor deterministic, nor do they execute code in the conventional sense. Their *computational mechanism* is chain-of-thought (CoT) reasoning, which does not map easily to any standard computational paradigm.

To study whether an LLM-powered AI agent can be a universal solver, we need more flexible foundations. (By *agent*, we generally refer to an LLM performing chain-of-thought (CoT) in order to solve a task, potentially with access to tools or an environment. These tools could be a Python executor, a proof checker, a web search, or another action to retrieve and process data, making the LLM an agent even if only the final action rendered affects the environment. For a given model and hardware, the computing cost of the agent roughly corresponds to the number of CoT tokens generated to find a solution.) In Section 2, we extend universal solvers from programs to general stochastic dynamical systems, allowing us to map the theory directly to LLMs with CoT. A key challenge is defining the *time that an LLM-powered agent needs to find a solution to a task*: naively using the expected length of CoT leads to degenerate values. We address this by introducing a new notion of *proper time* τ (Definition 2). This notion allows us to generalize Levin’s and Solomonoff’s results to general dynamical systems, showing in particular that LLMs can indeed power universal task solvers despite being unlike any Turing machine.

### 1.2. Intelligence Is About Time

Once we have secured the foundations for reasoning and computation, we turn to learning. Universal solvers of verifiable tasks are peculiar in that *no information needs to be learned to achieve universally optimal accuracy on any task*. For instance, to prove a theorem one could simply iterate over all possible proofs until a correct one appears, without ever having to study any math. Indeed, if we were to measure the information that a training dataset provides to such a task using classical notions (Shannon’s [4] or Kolmogorov’s [5]), we would find it to be null [6].

The role of learning in agentic AI is instead to identify statistical or algorithmic structures that make future inference *more time-efficient*. This suggests a notion, complementary to Shannon’s, that *time plays a crucial role in learning information*. In particular, we show that the optimal speed-up in finding a solution that a universal solver can achieve using past data is tightly related to the *algorithmic mutual information* [5] between the data and the solution:

**Theorem 1**  (Information is Speed)**.** *The maximum speed-up a task universal solver can achieve in finding an optimal solution h to a task from training on a dataset D is*

logspeed-up=I(h:D)

*where I(h:D) is the algorithmic mutual information between the data and the solution.*

We will give detailed definitions and proofs in the next sections; for now, we call attention to the fact that data can make a solver *exponentially faster*, consistent with our view that learning transduction can be equated to *amortizing inference computation*.

### 1.3. Scaling Laws for Speed

Having established that past data can speed up universal solvers, we examine how much speed-up is achievable as a function of the training dataset size. This requires making modeling assumptions about the data generating process or underlying mechanism.

A common assumption, including in Solomonoff’s work, is that real data, while complex on the surface, is generated through mechanisms or phenomena with low intrinsic complexity. This would intuitively suggest that there are ‘common reusable components’ we can learn from past data to help future reasoning. This intuition is, however, severely misleading, since the maximum speed-up obtainable by a solver is bounded by the complexity of the data generating distribution:

**Theorem 2** 
(Maximum Speed-up Bound)**.** *The maximum speed-up in an optimal solution h of a task sampled from a data generating process q—from which the training dataset D∼q is sampled— is*

logspeed-up≤K(q)

*where the Kolmogorov complexity K(q) is the length of the shortest program for q.*

Notably, if the data was generated by the Universal Prior (as in Solomonoff Induction [7,8]), there would be precisely nothing to learn (zero information). This challenges the fundamental assumption in generalization theory that *simplicity aids learning* [9]. While simplicity benefits *explainability*, it does not necessarily improve learning effectiveness. That simpler models generalize better is a consequence of how generalization is defined in the inductive setting; recent developments in LLMs have shown that simpler models are generally less effective at transduction.

From the results above, we see that the effectiveness of learning—and the asymptotic validity of scaling laws—hinges on the data generation process having effectively unbounded complexity. If complexity were bounded at K(q), scaling laws would plateau there; yet, empirically, we observe non-saturating power-law scaling [10,11]. This characteristic power-law trend is captured by Hilberg’s law for human-generated data [12,13]:

**Definition 1** 
(Hilberg Scaling)**.** *Let $X_{n}$ be a training dataset of n tokens and $Y_{n}$ be a test set of n tokens; then,*

$$I\left(X_{n}:Y_{n}\right)\propto n^{\beta }$$

*grows unbounded according to some distribution-specific rate β∈(0,1).*

We introduce a generalization of this conjecture for arbitrarily sized $X_{n}$ and $Y_{m}$ and prove the following **scaling law for speed after learning from data**:

**Theorem 3**  (Power Law of Inference vs Training)**.** *Let h be a chain-of-thought trajectory solving a task and let $T_{h}$ be its length. If the generalized Hilberg’s law holds, the log-speed-up from training on n tokens is*

$$\log\mathrm{speed}\text{-}\mathrm{up}\left(n\right)=T_{h}^{\beta }-\beta \frac{T_{h}}{n^{1-\beta }}$$

This result provides a *strong theoretical justification* for the empirically observed power-law scaling of inference time versus training time in reasoning LLMs [11] and can also be used to predict the scaling of space-bound LLMs (when the number of weights, rather than data, is the limit), thus providing guidance on how to scale resources when training universal solvers.

### 1.4. Inversion of Scaling Laws

The results described so far have dealt with the best model that *could* be learned from the data as we scale up. Empirically, models follow predicted power-law trends, suggesting near-optimal learning. But is this necessarily true? Is bigger always better?

Current scaling laws using prediction error (or perplexity) are sometimes used as a proxy for intelligence, arguing that more data, bigger models, and more computing will lead to “super-intelligence” [14]. But, counterintuitively, as models become more powerful, learning becomes unnecessary since the model can rely more on exhaustive computation rather than insights from learned structure in the data. As ordinary scaling proceeds, better and better performance on benchmarks may come with less and less insight, all the way to the limit where infinite resources allow solving any task by brute force without any learning. More precisely, emergence of “intelligence” (in the sense of *inter legere*, “to gather from among (the data)”) goes hand-in-hand with optimizing a solution under time constraints.

**Theorem 4** 
(Learning and Time Optimization)**.** *Without time penalties, optimal inference can be achieved brute-force without any learning or insight. Conversely, any system that optimizes time must learn at least I(h:D)=logspeed-up bits from past data.*

The results above reveal that plots of accuracy-versus-size, routinely used to predict progress towards “super-intelligence,” can be misleading. By ignoring the cost of time, they encourage *savantry* over *intelligence*. Intelligent behavior should instead be measured by *success rate per unit time/computing*. Properly accounting for the cost of time using the net reward, we see that an optimal agent balances time and accuracy rather than blindly maximizing reward through brute-force search. This optimal operating point depends on the task, the environment, and the particular user’s needs. Hence, it cannot be predetermined during training but rather must be calibrated and controlled by the user at deployment to fit the situation at hand.

The theorems above highlight a core tension: in inductive learning, achieving generalization relies on *minimizing* the complexity of the trained model—that is, the amount of information it shares with the specific training set—in keeping with information theoretic generalization bounds [15,16]. In universal solvers, by contrast, we seek to *maximize* the information the trained model acquires from the training data—within the limit of what the model can store and retrieve. Such information is beneficial because facts, patterns, or heuristics that were irrelevant to the training task may turn out to be handy when reasoning to solve future tasks.

### 1.5. Maximizing a Continuous Reward

Thus far we have focused on systems that need to reach a verifiable goal. More generally, a system may be trying to optimize a continuous reward function R(x,y). This introduces two challenges: we do not know when we have found the maximum, and we do not know if the increase in reward from further exploration will compensate the cost (which also depends on the user). Note that, while in reinforcement learning the solver is tasked with finding a policy for a particular environment (which may be partly unknown), here, we seek solvers that can operate in *any* environment, each with its own reward; when the reward function itself is unknown, we must again face the problem of learning it inductively.

If we have no way to estimate the predicted improvement in reward as a function of additional effort, tree search algorithms are worst-case optimal [17]. If, on the other hand, we can forecast future rewards [18], we show that the problem can be formalized as a Pandora’s box problem [19,20], which provides both an optimal search strategy and an optimal stopping rule.

In the next sections, we expand on the summary above in greater detail.

## 2. Dynamical Systems as Computation

Let us consider an example of an LLM solving a task using Chain-of-Thought (CoT):

**Example 1.** 
***What is the largest zero of*** $x^{3}+5x^{2}-74x+120$***?***

*I need to find the zeros of the polynomial $f\left(x\right)=x^{3}+5x^{2}-74x+120$. Let me try to factor this polynomial. First, I check if there are any rational roots using the rational root theorem.*
*The possible rational roots are the factors of 120 divided by the factors of 1, which means the possible rational roots are: ±1, ±2, ±3, ±4, ±5, ±6, ±8, ±10, ±12, ±15, ±20, ±24, ±30, ±40, ±60, ±120.*
*Let me test some of these: For x=1:1+5−74+120=52≠0. For x=−1:−1+5+74+120=198≠0. For x=2:8+20−148+120=0. ✓*
*So, x=2 is a root! This means (x−2) is a factor.*
*Now I perform polynomial division to find the other factor: $x^{3}+5x^{2}-74x+120=\left(x-2\right)\left(x^{2}+ax+b\right)$.*
*Expanding the right side: $\left(x-2\right)\left(x^{2}+ax+b\right)=x^{3}+ax^{2}+bx-2x^{2}-2ax-2b=x^{3}+\left(a-2\right)x^{2}+\left(b-2a\right)x-2b$.*

*Comparing coefficients:*

- *Coefficient of $x^{2}$: a−2=5, so a=7.*
- *Coefficient of x: b−2a=−74, so b−14=−74; thus. b=−60.*

*I continue factoring $x^{2}+7x-60$ by finding two numbers that multiply to −60 and add to 7. The numbers 12 and −5 work perfectly, since 12×(−5)=−60 and 12+(−5)=7. This means I can split the quadratic into (x+12)(x−5).*
*Combining all factors gives $x^{3}+5x^{2}-74x+120=\left(x-2\right)\left(x+12\right)\left(x-5\right)$, revealing the zeros at x=2, x=−12, and x=5. The largest zero is at 5.*

Clearly, this chain of thought is acting as some kind of computational device. However, it is nothing resembling a program since the results of the computation are intermixed with instructions (code) and comments, e.g.,

$$\mathrm{For}\;x=1,\underset{\mathrm{code}}{\underset{︸}{1+5-74+120}}=\underset{\mathrm{result}}{\underset{︸}{52}}$$

It is also not the trace of execution of an underlying algorithm since the code is generated dynamically based on the output of previous operations. Also, unlike standard programs, the particular CoT tokens are not necessarily meaningful; just outputting dots “…” may still lead to the correct result [21].

A more sound view is that an autoregressive LLM performing CoT is a stochastic dynamical system, where the prompt represents the initial state, the trained model backbone represents the transition probability, and each CoT trace is a sample trajectory. CoT *performs computation* in the sense that starting from an initial state, the system evolves until it reaches a *terminating* state (one where the network is confident it can answer), at which point it outputs a final answer.

Of course, it is well understood that a (deterministic or stochastic) dynamical system can solve computational tasks (Deterministic Finite Automata, Turing machines, Game of Life, etc.). But LLM systems are quite peculiar. They were not designed to solve a specific task but rather to be *universal solvers*: given a description of a task, the system should be able to find a solution. Moreover, rather than brute-forcing a solution, we expect it to find the fastest way to solve the problem, as well as access past information (e.g., the “rational root theorem” in the example above) to significantly speed up the solution.

### 2.1. Notation

In this section we introduce the notation used throughout this paper. (For those with a background in dynamical system theory, in Appendix B, we map the notation to one more reminiscent of continuous dynamical systems). Let s∈S be a state in a potentially infinite state space S and let t∈N be a time index. A sequence of states $h=(s_{1},\ldots ,s_{n})$ is called a trajectory or path. Its length is the time T(h)=n. A stochastic dynamical system is defined by the transition probability $\nu (s_{t+1}|s_{t})$. We say that *h* is a trajectory between two states u,v∈S if $s_{1}=u$ and $s_{n}=v$. The probability $\nu \left(h\right)=\prod_{t=1}^{n-1}\nu \left(s_{t+1}|s_{t}\right)$ of a trajectory is the product of the transition probabilities along the path.

The system should be able to read inputs and output answers. Let Σ be the input/output alphabet. We assume that the system has a set F⊂S of *terminating* states and a function $\mathrm{dec}:F\to \Sigma^{*}$ that, given a terminal state, generates an output. We also assume that there is a function $\mathrm{enc}:\Sigma^{*}\to S$ that encodes the input into a state of the dynamical system, where $\Sigma^{*}$ is the set of all possible finite trajectories. We say that a trajectory $h=(h_{1},\ldots ,h_{t})$ terminates with output *a*, which we write as h⇒a—if $h_{t}\in F$ and $\mathrm{dec}(h_{t})=a$.

Let $x\in \Sigma^{*}$ be an input. For simplicity, we assume that all trajectories starting from enc(x) and ending in a terminating state terminate with the same output, or with a special <error> token. This allows us to write ν(x⇒a), meaning the dynamical system ν starting from enc(x) terminates with *a*. While this is generally restrictive, we mainly study settings where the answer is verifiable, in which case we can trivially return error if the output is not correct. (A more standard and less restrictive definition is to ask that the answer is correct at better than chance level; that is, we would say ν(x⇒a) if $P[h\Rightarrow a|h_{1}=\mathrm{enc}\left(x\right)]>2/3$).

**Example 2.** 
*Two key systems we are interested in are as follows:*

***LLMs.** The state is the set of activations of the LLM after reading some tokens. This is the set of activations (known as the key-value (KV) cache) for an autoregressive Transformer, or more generally the hidden state for a State Space Model. The transition function $\nu (s_{t+1}|s_{t})$ generates the next token given the state $s_{t}$ and uses it as input to generate state $s_{t+1}$. The final states are the states at which the LLM outputs an* *<end_of_thought>* *token. The decoding function consists of letting the network generate the answer after* *<end_of_thought>* *. The encoding function simply lets the LLM read the input tokens to update its initial state.*

***Turing Machines.*** *The state of a Turing machine is the content of its tape at a given time, plus its internal state. The transition function $\nu (s_{t+1}|s_{t})$ updates the tape and its internal state as usual, either deterministically in a standard Turing machine or randomly in a probabilistic machine.*

We also make use of several notions from algorithmic information theory [5]. Let *x* be a string, its *Kolmogorov Complexity* K(x) is defined as the length (in bits) of the shortest program that terminates outputting *x*. Given two strings *x* and *y*, their *algorithmic mutual information* is I(x:y):=K(x)+K(y)−K(x,y)=K(x)−K(x|y) (up to logarithmic additive terms). This can be interpreted as how much more *x* can be compressed if we have already observed *y*. Recall that, by Shannon’s Coding Theorem, given any probability distribution ν(x) over binary strings, there is a corresponding encoding algorithm that encodes a string *x* in $\ell_{\nu }\left(x\right):=-\log_{2}\nu \left(x\right)$ bits.

### 2.2. Proper Time

As we have anticipated, transductive learning is about solving generic tasks quickly. But how do we define the *time* that a *stochastic* system needs to find a solution to the task? The question is subtle, since if we look at the length of a particular sampled trajectory, randomness can make an algorithm look arbitrarily faster or slower, without changing what it effectively computes.

Let us first consider a motivating example. Let f(x) be a function that is easy to evaluate, but can be inverted only through brute-force search (i.e., a ‘one-way function’). Given *y*, the task is to find a binary string *x* of length |x|=n such that y=f(x). A *deterministic* Turing machine must try all $2^{n}$ candidates for *x*, so the total expected time is $T=2^{n-1}$. On the other hand, a *stochastic* machine can *guess* the first *k* of *x*, and brute-force the remaining n−k, so every terminating trajectory has length $T=2^{n-k}$, but occurs with probability only $\nu \left(h\right)=2^{-k}$.


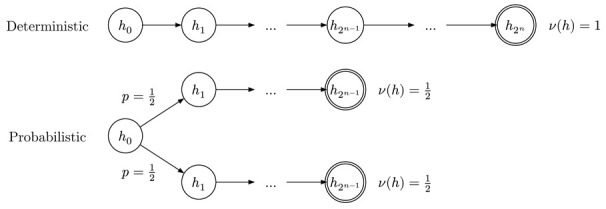


The probabilistic machine can then be arbitrarily faster than the deterministic machine as measured by the trajectory length T(h) even if, effectively, both are doing the same brute force search. If, however, we consider the ratio $\tau \left(h\right)=T\left(h\right)/\nu \left(h\right)=2^{n}$, we see that it remains constant, no matter how we branch the computation path: randomness shortens paths but also makes them rarer. This invariance suggests the following definition of “proper” time for single-trajectory targets.

**Definition 2** 
(Proper Time)**.** *Let $\nu (h_{t+1}|h_{t})$ be a stochastic dynamical system. Define the* proper time *to reach v from u as*

$$\tau_{\nu }\left(u\to v\right)=\min_{h_{u\to v}}\frac{T(h)}{\nu (h|u)}$$

*where the minimum is over all trajectories h from u to v, or $\tau_{\nu }=\infty$ if no trajectories exist. For an input–output specification, the proper time to terminate from input x with output a is*

$$\tau_{\nu }\left(x\Rightarrow a\right)=\min_{h\Rightarrow a\;\mathrm{and}\;h_{1}=\mathrm{enc}\left(x\right)}\frac{T(h)}{\nu (h|\mathrm{enc}(x))},$$

*where the minimum is over trajectories starting from enc(x) and terminating with output a.*

This definition is closely related to Levin Complexity [22] and its extension to tree search [17]. If the system is deterministic, then ν(h|u)=1 for any path, and $\tau_{\nu }$ reduces to standard running time. Conversely, we now show that $\tau_{\nu }$ indeed captures the actual computational effort required by a stochastic dynamical system when simulated deterministically.

**Theorem 5** 
(Dynamical Systems ⇒ Turing machines with same τ)**.**  *Let $\nu (s_{t+1}|s_{t})$ be a dynamical system. There is a deterministic Turing machine $M_{\nu }$, with access to an oracle to compute ν(·|·), such that*

$$T_{M_{\nu }}\left(x\Rightarrow a\right)\leq 2\;\tau_{\nu }\left(x\Rightarrow a\right)$$

The theorem follows directly by taking $M_{\nu }$ to be the Turing machine that implements the algorithm in the following key lemma:

**Lemma 1** 
(Levin Tree Search [17])**.** *Let u,v be two states. There is a deterministic algorithm A that discovers a path between them (if it exists) while visiting at most T nodes, where*

$$T=\tau_{\nu }\left(u\to v\right)$$

**Proof sketch.**  We sketch the argument underlying Levin-Tree Search [17]. For a partial trajectory (search-tree node) $h=(u=s_{0},s_{1},\ldots ,s_{t})$, we define its Levin cost:

$$\tau_{\nu }\left(h\right)\;:=\;\frac{\ell (h)}{\nu (h|u)}\;,\;\nu \left(h|u\right)=\prod_{i=0}^{t-1}\nu \left(s_{i+1}|s_{i}\right)\;,\;\ell \left(h\right)=t.$$

The algorithm maintains a frontier of unexpanded prefixes and repeatedly expands the prefix having minimum $\tau_{\nu }$. Along any root-to-leaf branch, $\tau_{\nu }$ is nondecreasing (depth increases while probability only decreases), which implies a best-first property: all prefixes with $\tau_{\nu }\leq c$ are expanded before any prefix with $\tau_{\nu }>c$, where $c:=\min_{h:u\to v}\tau_{\nu }\left(h\right)$ is the optimal goal cost. Let $T_{c}$ be the (finite) search tree consisting of all expanded nodes at the moment the first goal node is expanded. Every leaf *h* of $T_{c}$ satisfies $\tau_{\nu }\left(h\right)\leq c$; hence, ℓ(h)≤cν(h∣u). Moreover, the number of expanded nodes is at most the sum of the depths of leaves, $|N\left(T_{c}\right)|\leq \sum_{h\in L(T_{c})}\ell \left(h\right)$, since each leaf contributes at most one count to each of its ancestors. Therefore,

$$|N\left(T_{c}\right)|\;\leq \;\sum_{h\in L(T_{c})}\ell \left(h\right)\;\leq \;c\sum_{h\in L(T_{c})}\nu \left(h|u\right)\;\leq \;c,$$

assuming that leaf probabilities in a prefix tree sum to at most 1. Thus, Levin Tree Search reaches *v* after at most $c=\min_{h:u\to v}\ell \left(h\right)/\nu \left(h|u\right)$ node expansions. □

Since all computation today is executed on deterministic logic hardware, Theorem 5 validates $\tau_{\nu }$ as the “proper” way to measure time for a stochastic dynamical system. (The name has a loose analogy with relativistic *proper time*: like $\tau =t^{2}-x^{2}$, our $\log\tau_{v}=T-\log v\left(h\right)$ mixes temporal and probabilistic ‘distances,’ providing a representation-invariant clock). It also frames τ as a fundamental property of the algorithm we are executing, rather than a function of the stochasticity of its implementation.

**Remark 1.** 
*It is useful to compare proper time with other candidate measures of computational cost. Consider a system that, with high probability 1−ϵ, terminates in a short number $T_{0}$ of steps but, with small probability ϵ, and enters an infinite, non-terminating, chain where every transition has probability $\nu (s_{i+1}|s_{i})=1$. If we sample a random trajectory, the expected time E[T] is infinite: even though the non-terminating trajectory is entered with low probability, its infinite length dominates the expectation. (The same conclusion holds even when all trajectories are forced to terminate. For example, suppose a system has probability $p_{n}=\epsilon \;2^{-n}$ of entering a trajectory of length $T_{n}=2^{n}$. Then, $E\left[T\right]=\left(1-\epsilon \right)\;T_{0}+\epsilon \sum_{n}1=\infty$). Likewise, the Levin-style quantity $E\;\left[\frac{T(h)}{\nu (h|u)}\right]$ diverges: along the non-terminating chain, the trajectory probability stays bounded away from zero (since each transition has probability one), yet the time grows without bound, so their ratio diverges.*

*The proper time $\tau_{\nu }\left(x\Rightarrow a\right)=\frac{T_{0}}{1-\epsilon }$, by contrast, remains bounded and close to $T_{0}$. Intuitively, proper time discounts trajectories by their probability of being reached, rather than conditioning on having entered them, and so it is not held hostage by low-probability pathological paths.*

*This distinction has a practical consequence: to harness the computational power of a stochastic system, it is not enough to sample trajectories from it. Instead, a dovetailing strategy, as in Lemma 1, should be used (in the next section, we also show that a sampling approach with a suitable restart schedule can achieve close to optimal time). More broadly, this highlights that a standalone LLM is not itself an optimal solver since its time to terminate can be arbitrarily large. Rather, an optimal solver agent is constructed from the LLM by wrapping it in an appropriate search procedure. (This construction requires the ability to restart or roll back trajectories, a property naturally satisfied by today’s dominant agentic settings—code execution with unit tests, theorem proving with proof checkers, and tool-augmented reasoning in sandboxed environments—where the agent’s actions do not have irreversible side-effects on the external world).*

*Note that using Levin Tree Search increases the memory requirements compared to the original stochastic system since we have to explore multiple states in parallel. The total amount of states to keep in memory is bounded by the proper time τ itself (Lemma 1), and the bound is tight for a shallow tree with a high branching factor. However, note that time guarantees similar to Levin Tree Search can be obtained without increased memory usage by, for example, using sampling with a restart schedule.*

In a deterministic system, the time (path length) between states is a distance. Similarly, the following theorem establishes that for a path x→y→z, the proper time to go from x→z cannot be greater than the time it takes to first go to *y* and then to *z*. It will play an important role in multiple proofs.

**Lemma 2** 
(Proper Time is submultiplicative)**.** *Let x,y,z be three states. Then,*

$$\tau_{\nu }\left(x\to z\right)\leq \tau_{\nu }\left(x\to y\right)\cdot \tau_{\nu }\left(y\to z\right)$$

**Proof.**  Let $h_{x\to y}$ and $h_{y\to z}$ be paths that realize the minimum in the definition of τ. We can construct the path $h_{x\to z}=h_{x\to y}\circ h_{y\to z}$ composing the two paths. By Definition 2, we have

(1) $$\begin{array}{cc} \tau_{\nu }\left(x\to z\right) & \leq \frac{T(h_{x\to z})}{\nu (h_{x\to z})}=\frac{T\left(h_{x\to y}\right)+T\left(h_{y\to z}\right)}{\nu \left(h_{x\to y}\right)\nu \left(h_{y\to z}\right)} \end{array}$$

(2) $$\begin{array}{cc} \; & \leq \frac{T\left(h_{x\to y}\right)\;T\left(h_{y\to z}\right)}{\nu \left(h_{x\to y}\right)\;\nu \left(h_{y\to z}\right)} \end{array}$$

(3) $$\begin{array}{cc} \; & =\tau_{\nu }\left(x\to y\right)\;\tau_{\nu }\left(y\to z\right) \end{array}$$

where in the second line (2), we used the fact that $T_{1}+T_{2}\leq T_{1}T_{2}$ whenever $T_{1}\geq 2$ and $T_{2}\geq 2$, which is automatically satisfied when the states are distinct. If two or more states are the same, the property can be easily checked by hand. □

This also implies that logτ(x→z)≤logτ(x→y)+logτ(y→z), which makes logτ an *asymmetric distance* between states. Note that τ is sub-multiplicative, while deterministic time is sub-*additive*. This is because in a stochastic system, time may be dominated by the time to find a suitable combination of paths, and probability of the composition of two paths is a product.

These two lemmas suffice to prove the key theorems in the rest of this work, including providing a straightforward construction for a generalization of Solomonoff–Levin Universal Search Algorithm [2,3].

### 2.3. Multiple Successful Paths

The quantity in Definition 2 measures the cost to *uncover a particular trajectory*. Many tasks, however, accept *any* trajectory leading to one of the final states s∈F. In that setting, multiple distinct paths can succeed, and the right notion aggregates their probabilities. Let

$$F_{\nu }\left(t;F\right):=\underset{\nu }{\mathrm{Pr}}\left(\mathrm{reach}\;F\;\mathrm{within}\;t\;\mathrm{steps}\right)$$

be the *success-by-time* curve. If we run independent trials of length *t* (restarting after *t* steps), we need in expectation $1/F_{\nu }\left(t;F\right)$ trials for one success, for total expected work $t/F_{\nu }\left(t;F\right)$. Optimizing over the cutoff gives a canonical baseline:

$$\tau_{\nu }^{*}\left(F\right):=\underset{t\geq 1}{\inf}\;\frac{t}{F_{\nu }\left(t;F\right)}.$$

This general notion (i) strictly improves over any single-path bound when many solutions exist, and (ii) collapses to the proper time when there is effectively one successful path. In principle, to simulate the stochastic system in total time $\tau_{\nu }^{*}\left(F\right)$, we would need the unknown optimal cutoff *t*. However, universal Luby-type restart schedules [17,23] achieve expected work within a logarithmic factor of the optimum fixed-cutoff policy:

$$\mathrm{Expected}\;\mathrm{work}\;=\;O\left(\tau_{\nu }^{*}\left(F\right)\;\log\tau_{\nu }^{*}\left(F\right)\right).$$

Thus, $\tau_{\nu }^{*}\left(F\right)$ characterizes intrinsic difficulty ‘up to logs.’ For clarity of exposition, in the rest of this paper, we focus on $\tau_{\nu }\left(h\right)$, but all results extend naturally to $\tau_{\nu }^{*}\left(F\right)$.

### 2.4. Universal Dynamical Systems

A key part of defining a computation system is the existence of universal systems. Generally, we want a system in the class to be able to simulate any other system in the same class. However, since time is a key quantity for us, we need to ensure the time to simulate is similar to the original time.

**Definition 3** 
(Linear-Time Universal Dynamical System)**.** *Let ν be a dynamical system and let [ν] be its encoding. We say that dynamical system U is linear-time universal if for any ν, we have*

$$U\left(x\left[\nu \right]\Rightarrow a\right)\Leftrightarrow \nu \left(x\Rightarrow a\right)\;\mathrm{and}\;\tau_{U}\left(x\Rightarrow a\right)\leq C_{\nu }\tau_{\nu }\left(x\Rightarrow a\right)$$

*for some constant $C_{\nu }$ that depends on ν but not on the input x.*

Since for any dynamical system ν there is a Turing machine $M_{\nu }$ which simulates ν in the same proper time, to satisfy the definition it is enough to verify that for any Turing machine *M*, we have

(4) $$U\left(x\left[M_{\nu }\right]\Rightarrow a\right)\Leftrightarrow M\left(x\right)=a\;\mathrm{and}\;\tau_{U}\left(x\Rightarrow a\right)\leq C_{M}T_{M}\left(x\Rightarrow a\right)$$

which is generally easier to verify. This observation trivially gives us the following.

**Corollary 1.** 
*There exists a Linear-Time Universal Dynamical System.*

**Proof.**  Any linear-time universal Turing machine is a dynamical system and by definition can emulate other Turing machines in linear time. □

## 3. Universal Solvers

We are now finally ready to introduce universal solvers, which are our main focus. A universal solver is a dynamical system that can efficiently find a solution to an arbitrary problem, if one exists. We formalize it as follows.

Let f(x,y):X×Y→{0,1} be a computable function. We say that *y* is a witness of *x* if f(x,y)=1. A universal search program is any program *S* that, provided with an oracle for *f* and an input *x*, terminates with outputting *y* such that f(x,y)=1 (we generalize this to continuous rewards in Section 6):

S(x⇒y)⇔f(x,y)=1

If *y* does not exist, the program is allowed to terminate with an error or not terminate at all. Generally, together with the input *x*, we may also pass a description of the objective f(x,y) so that the search program is not blind. To keep the notation uncluttered, we do not denote this additional input.

It is always possible to find a witness to any problem by just enumerating all possible *y* in a dovetail fashion and checking for f(x,y)=1 using the oracle for *f*. However, we are interested in search programs that are as efficient as possible.

**Definition 4** 
(Universal Solver)**.** *A dynamical system U is a* universal solver *system if for any objective f(x,y) and any other system A that solves the problem—i.e., for all x, A(x⇒y) with f(x,y)=1—we have*

$$\tau_{U}\left(x\Rightarrow y\right)\leq C_{A}\tau_{A}\left(x\Rightarrow y\right).$$

That is, for any task, a universal solver is at most a constant factor slower than the best possible system *A* that solves that particular task. The existence of a universal solver is non-trivial. Levin introduced the notion of universal search, as well as a sketch of the existence of such a system in the same paper that introduced the notion of NP-Complete problems [2]. Solomonoff later realized its importance for machine learning and provided a detailed proof [3]. With the formalism we introduced, the existence proof is straightforward and can be generalized to any stochastic system, with Turing machines as a special case.

**Theorem 6** 
(Existence of Dynamical System Universal Solvers)**.** *Let m be any distribution encoding programs from which we can sample. Then, there is a dynamical system $U_{m}$ such that for any solver A,*

$$\tau_{U_{m}}\left(x\Rightarrow y\right)\leq C_{A}^{\prime }2^{-\log m(A)}\tau_{A}\left(x\Rightarrow y\right).$$

*In particular, $U_{m}$ is a universal solver with constant $C_{A}=C_{A}^{\prime }2^{-\log m(A)}$.*

**Proof.**  Let *U* be any linear-time universal system as in Definition 3. Construct a composite dynamical system $U_{m}$ as follows. First, given *x*, use *m* to sample a program encoding, call it [A], and append it to the input to get x[A]. Then, run the universal system *U* to execute [A] on *x*.

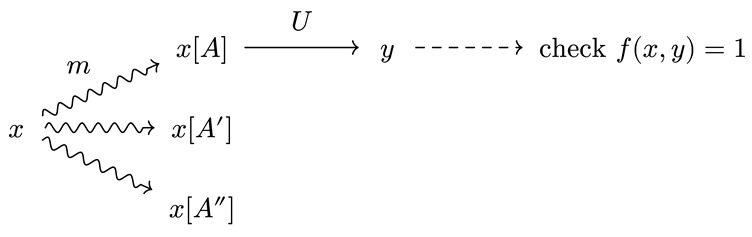

Let *A* be any algorithm that solves the search problem. Then, U(x[A]⇒y), giving us a path x→x[A]→y from the input *x* to the solution *y*. Applying the submultiplicativity of τ to this path from Lemma 2, and using the definition of universal dynamical system (Definition 3), we have

(5) $$\begin{array}{cc} \tau_{U_{m}}\left(x\Rightarrow y\right) & \leq \tau_{m}\left(x\to x\left[A\right]\right)\tau_{U}\left(x\left[A\right]\Rightarrow y\right) \end{array}$$

(6) $$\begin{array}{cc} \; & \leq C_{A}^{\prime }\tau_{m}\left(x\to x\left[A\right]\right)\tau_{A}\left(x\Rightarrow y\right) \end{array}$$

Note that by definition of $U_{m}$ we have $\tau_{U_{m}}\left(x\to x\left[A\right]\right)=T/m\left(A\right)=1/m\left(A\right)$ (where we assume that the entire program [A] is sampled in one step). Replacing this identity in the above, we get

$$\tau_{U_{m}}\left(x\Rightarrow y\right)\leq C_{A}^{\prime }2^{-\log m[A]}\tau_{A}\left(x\Rightarrow y\right)$$

which gives the desired time bound. □

The construction above instantiates a particular universal solver that first ‘guesses’ a program that may solve the task and then executes it. Of course, in general, universal solvers need not be a one-shot guess: human problem-solvers will not blindly guess an algorithm and execute it but will rather interleave partial computations, observations, backtracking and shortcuts. Our general stochastic dynamical-system view already subsumes such interactive behavior. Nonetheless, the search algorithm presented is universal (as in, no other can be significantly faster) and already allows us to make some general observations:

**Speed of a universal solver.** For any solver *A* that succeeds on *x*, the universal solver above solves the problem in time:

$$\tau_{U_{m}}\left(x\Rightarrow y\right)\;\leq \;C_{A}\;2^{-\log m(A|x)}\;\tau_{A}\left(x\Rightarrow y\right)$$

That is, the slowdown with respect to an arbitraty solver is the simulation constant $C_{A}$ times the inverse prior weight of the right program. This bound highlights two levers for learning. The term $2^{-\log m(A|x)}=2^{\ell_{m}\left(A|x\right)}$ depends on the code length $\ell_{m}$: if tasks reuse a small set of subroutines, reshaping *m* to give these short codes yields exponential gains (we return to this in Section 4). The factor $C_{A}$ reflects how many steps our base dynamics spend simulating a single step of *A*; when particular transition patterns recur, we can ‘macro-step’ them—effectively adding shortcuts in the dynamics—to shrink $C_{A}$. These ideas extend beyond the guess–execute prototype to any universal search system. We will study them in detail in later sections.

**Universal Search and Universal Computation.** From the proof of Theorem 6, we see that starting from a universal system, we can easily construct a time-optimal universal search system. The following straightforward theorem shows that the converse also holds. For a system to be a universal solver, it needs to be a universal computation system.

**Theorem 7** 
(Universal Search ⇒ Linear-Time Universal System)**.** *Let U be an optimal universal search program. Then, it is also a universal dynamical system.*

**Proof.**  Let *M* be a Turing machine. Construct the function f(x,y)=1 if M(x)=y and 0 otherwise. By the definition of optimal universal search, we have U(x[f])⇒y⇔f(x,y)=1, which implies M(x)=y by construction. Moreover,

(7) $$\begin{array}{cc} \tau_{U}\left(x\right) & \leq C_{M}\tau_{M}\left(x\to x\left[M\right]\right)T_{M}\left(x\right) \end{array}$$

(8) $$\begin{array}{cc} \; & =C_{M}^{\prime }T_{M}\left(x\right); \end{array}$$

therefore, it is linear-time universal. □

The claim is straightforward but it has an important implication: *if we train a model to solve a sufficiently general set of tasks, the model will necessarily learn to simulate a universal Turing machine*. Whether this has already happened in the current generation of LLMs pretrained on language has been hotly debated. Depending on the exact setting and access to tools or external memory, one can argue one way [24,25,26] or the opposite [27,28]. Our result establishes beyond debate what is possible.

**Optimality through meta-learning.** An important property of a universal search system is that it is necessarily optimal, meaning that no other universal search system can be arbitrarily faster:

**Lemma 3.** 
*Let U and $U^{\prime }$ be two universal search systems. Then, for any task f, we have*

$$\tau_{U}\left(x\Rightarrow y\right)\leq C_{U^{\prime }}\tau_{U^{\prime }}\left(x\Rightarrow y\right).$$

*where the constant $C_{U^{\prime }}$ does not depend on task f or the input x.*

**Proof.**  Since by universality $U^{\prime }$ finds the solution to the task *f*, we can take $A=U^{\prime }$ in the definition of universal search, giving us

$$\tau_{U}\left(x\Rightarrow y\right)\leq C_{U^{\prime }}\tau_{U^{\prime }}\left(x\Rightarrow y\right)$$

Hence, *U* is at most $C_{U^{\prime }}$ times slower than $U^{\prime }$, where $C_{U^{\prime }}$ does not depend on the task *f*. □

The proof is a trivial manipulation of the definitions, but it underlies a key concept that in modern terminology would be called *meta-learning*. Let us use the particular universal system in Theorem 6 to make the point explicit. For this, the time required to solve a task depends on −logm(A), the encoding length of its optimal solution. It is *a priori* possible that a system $U^{\prime }$ may achieve a better time on some tasks by learning a better encoding $m^{\prime }$ specific for them. However, *U* can just search (meta-learn) the solver $U^{\prime }$ and use it to solve the task, leading to a slow down of at most $2^{-\log m(U^{\prime })}$. In practice, the constant $2^{-\log m(U^{\prime })}$ is too large, and we need to amortize it through learning, which is our focus for most of this work.

### Universal Solvers and Sampling

By Theorem 5, we can convert a stochastic system ν to a deterministic program that finds a solution in time $T=\tau_{\nu }\left(x\mapsto y\right)$. However, this program is *not* obtained by naively sampling a random trajectory up to completion, as one may be tempted to do. In fact, doing that would have an infinite expected runtime:

**Example 3.** 
*Let ν be any computable prior that gives non-zero mass to all programs (e.g., the Universal Prior); then, $E_{A∼\nu }\left[T_{A}\right]=\sum \nu \left(A\right)T_{A}=\infty$, even assuming that we have an oracle preventing us from running algorithms that do not terminate. To see this, consider the program A that computes ν(A) and runs for $\nu {\left(A\right)}^{-1}$ steps before terminating. Then, ν(A)T(A)=1 and there are infinite such programs in the expectation.*

This highlights an important principle: if we have a way to guess a possible solution, in general it is not time-optimal to keep generating guesses and testing them. For an LLM, this means that sampling CoT traces until one succeeds is not a good idea. Rather, we need to keep open multiple possibilities and smartly allocate a time budget between all of them. To add some color, imagine trying to prove a theorem. You will likely start with the most likely guess, but if it starts to take too long with no solution in sight, you will try spending some time on another approach and perhaps come back to the original approach later.

The construction in Theorem 5 which achieves τ on a deterministic system can be made into a stochastic algorithm. The algorithm above hinges on keeping multiple hypotheses at the same time and continuing to explore them with increasingly more budget. What prevents us from having a system that achieves the same expected time by sampling individual trajectories?

We have seen before that such a system cannot sample programs directly from ν(A) as the expected time could easily be infinite. A good guess is that we need to sample from the distribution (this distribution is closely related to Schmidhuber’s Speed Prior [29] and Filan et al.’s $S_{\mathrm{Kt}}$ prior [30]):

(9) $$\nu_{t}\left(A\right)=\frac{1}{Z}\frac{\nu (A)}{T(A)},$$

where *Z* is the normalization constant, which prioritizes programs that have a short running time. This is indeed the case.

**Theorem 8** 
(Time-Weighted Sampling)**.** *Let ν be a universal search system. If we sample trajectories from*

(10) $$h∼\nu_{t}\left(h|u\right)\propto \frac{\nu (h|u)}{T(h)}$$

*and run them to completion, the total amount of operations we need to perform before finding a solution is*

(11) $$E\left[T_{\mathrm{total}}\right]=\tau_{\nu }\left(x\mapsto y\right).$$

**Proof.**  Let $h_{*}$ be a trajectory solving the task and let $n_{*}$ denote the number of iterations before $h^{*}$ is sampled. In expectation, we have $E\left[n_{*}\right]=\frac{1}{\nu_{t}\left(A\right)}=Z\frac{T(h)}{\nu (h)}$. We now need to compute how much time is spent validating each of the $n_{*}$ samples. The expected time that we need to spend validating a single sample from $\nu_{t}$ is

(12) $$E_{A∼\nu_{t}}\left[T_{A}\right]=\frac{1}{Z}\sum_{i}\frac{\nu (A_{i})}{T_{A_{i}}}T_{A_{i}}=\frac{1}{Z}$$

so the total time we need to spend validating the $n_{*}$ is

(13) $$T_{\mathrm{total}}=E\left[T_{1}+T_{2}+\ldots +T_{n^{*}}\right]=E\left[n_{*}\right]E\left[T_{i}\right]=Z\frac{T(A)}{\nu (A)}\frac{1}{Z}=\frac{T(A)}{\nu (A)}$$

which gives the desired result. □

Hence, a universal search algorithm that only wants to consider one guess at the time has to learn how to sample from $\nu_{t}\left(h\right)$, which means that in addition to estimating the probability ν(h) that a solution is correct, it should also be able to predict the time T(h) that it will take to run it.

The distribution $\nu_{t}$ is actually computable (the time T(h) may be undecidable, but to upper bound $\nu_{t}\left(h\right)$ within ϵ, we just need to show that ν(h)/T(h)<ϵ and, hence, run for T=ν(h)/ϵ steps). However, in [30], it is shown that $\nu_{t}\left(h\right)$ takes double exponential time in 1/ϵ to approximate, and this essentially requires running multiple programs, which we want to avoid in the first place.

Hence, the only option left if we want to avoid searching over trajectories is to *train* a system to approximate both the likelihood of the solution and the cost of time. While this will not be our focus, the following importance weighted training scheme gives a way to train.

**Theorem 9** 
(Importance-Weighted Training)**.** Let ν be a dynamical system. Let $h=(h^{1},\ldots ,h^{n})$ be a batch of trajectories sampled from ν. Then, the distribution $\mu^{*}$ minimizing

(14) $$\mu^{*}=\arg\min_{\mu }E_{h}\left[\sum_{i}w_{i}\mu \left(h^{i}\right)\right]\;\mathrm{with}\;w_{i}=\frac{T(h^{i})}{\sum_{j}T\left(h^{j}\right)}$$

is exactly $\mu^{*}=\nu_{t}$.

## 4. Scaling Laws for Speed

By the definition of a universal solver, given a function f(x,y) and an input *x*, there is a trajectory *h* finding a witness *x* if such a witness exists. Let *h* be the shortest such trajectory, i.e., the one with minimal T(h). The total time required by the universal search system to find it is

(15) $$\tau_{U}=2^{-\log\nu (h)}T\left(h\right)=2^{\ell_{\nu }\left(h\right)}T\left(h\right)$$

where we define $\ell_{\nu }\left(h\right)=-\log\nu \left(h\right)$ to be the compression length of the trajectory using ν. How do we reduce the search time $\tau_{U}$? We could reduce the thinking time T(h) by learning to skip some steps to get directly to the solution. But the largest improvement will come from reducing the exponential factor $2^{\ell_{\nu }\left(h\right)}$. This is the time needed to *guess* the correct solution to the problem. Thanks to Shannon’s Coding Theorem, we can improve the probability of guessing the solution, thus speeding up the search, by instead finding a way to *reduce the compression length* of *h*. We can do this by learning from a dataset *D*.

For example, suppose we have a list of programs that have worked well in the past. If we notice that some pieces of code tend to appear frequently (say, the code to compute an FFT), we could change the encoding to replace those pieces of code with a unique name. This reduces the length of those programs, making them more likely to be sampled. Not only that, but any program reusing those components is more likely to be guessed in the future.

Another example to add color: Suppose that while proving theorems, we often use the same sequence of steps. We probably will want to turn it into a named theorem—e.g., “Cauchy–Schwarz inequality”—which will also make us more likely to try to use it in future problems. In this spirit, let us crystallize this in the following:

**Theorem 10** 
(Better compression ⇔ Faster search)**.** *For a universal search system with model ν, improving the compression of a trajectory h by* Δ *bits accelerates its discovery by a factor of $2^{\Delta }$.*

Let us now formalize what learning from data means. Given some data *D*, we denote by

(16) $$\ell_{\nu }\left(h|D\right)=-\log\nu \left(h|D\right)$$

the negative log-likelihood given by the model to a trajectory after observing dataset *D*. One possibility is that we *train* on data *D*. In this case, we assume that $\nu_{\theta }$ is a parametrized family of distributions. Let $\theta_{D}$ be the parameters obtained after training on *D*. Then, we define $\nu \left(h|D\right):=\nu_{\theta_{D}}\left(h\right)$ as the likelihood given to *h* by the trained model. Alternatively, we can do *in-context learning* (ICL) or *prompting* where we feed data *D* to the model to obtain state $s_{D}$, and then we set $\nu \left(h|D\right):=\nu \left(h|s_{D}\right)$ the likelihood of the trajectory after having seen the data. It could also be that the model ν(h) has a way to retrieve information from *D*, a process known as *retrieval-augmented generation* (RAG). Any mix of these methods may be used (some data is used to train, some to prompt, and some for retrieval). While different in implementation, from a theoretical perspective, there is no fundamental difference between these ways of using past data, which is why we can write generically ν(h|D).

In our setting, the benefit of learning is not measured by better accuracy—since we have a verifier, sooner or later, we *will* find a correct solution—but rather by the reduction in search time. The speed-up factor achieved after training on the data is given by the ratio

(17) $$\frac{\tau_{\nu }\left(h\right)}{\tau_{\nu_{D}}\left(h\right)}=2^{\ell_{\nu }\left(h\right)-\ell_{\nu }\left(h|D\right)}=2^{I_{\nu }\left(h:D\right)}$$

where we define the ν-algorithmic mutual information:

(18) $$I_{\nu }\left(h:D\right)=\ell_{\nu }\left(h\right)-\ell_{\nu }\left(h|D\right)$$

So, the speed up of universal search is given by the algorithmic mutual information between inference time trajectories and past data. We highlight this in the following.

**Theorem 11** 
(Information is speed)**.** *The log-speedup of a search algorithm after seeing data D is*

(19) $$\log\frac{\tau_{\nu }\left(h\right)}{\tau_{\nu }\left(h|D\right)}=I_{\nu }\left(h:D\right).$$

**Remark 2.** 
*Theorem 11 follows easily from the straightforward algebraic manipulation in Equation (17). This is possible thanks to the work we already did in defining and validating the notion proper time, which connects the likelihood of trajectories in a dynamical system (which relates to information) with the time necessary for the system to find a solution*.

We are interested in ν representing very good compressors (since we want to minimize $\ell_{\nu }\left(h\right)$). Asymptotically, the best compressor is the universal prior $m\left(h\right)\propto 2^{-K(h)}$, for which $I_{m}\left(X:Y\right)$ becomes *the* algorithmic mutual information [5]:

(20) I(h:D)=K(h)−K(h|D)=K(h)+K(D)−K(Dh).

While we are interested in $I_{\nu }\left(h:D\right)$, we can use I(h:D) as a proxy of what is the best we could achieve asymptotically. The advantage is that I(h:D) has a number of theoretical properties that make it easier to work with.

The key question now is as follows: What is the maximum possible log-speedup I(h:D) we can get from learning? As it turns out, the answer is not straightforward and depends on some key assumptions about how real data works. Let us get there step by step.

The trajectory *h* is the trajectory of an optimal solution to a task (e.g., the optimal CoT to get to a solution, or the shortest execution trace of a program that solves the problem). Meanwhile, $D=\{h_{1},\ldots ,h_{n}\}$ is presumably created by collecting examples of trajectories that optimally solved tasks in the past.

A first guess (often done in the Minimum Description Length literature) is that solutions to real world problems tend to have low complexity. It therefore may make sense to hypothesize that $h_{i}∼m\left(h\right)=2^{-K(h)}$ is sampled from the universal prior itself, which favors low-complexity solutions. What would be I(h:D) in this setting? Disappointingly, we can show that

(21) $$P\left[I\left(h:D\right)>k\right]\leq nc2^{-k}$$

so the probability that past data *D* share substantial information with the solution to the present task *h* and therefore can lead to substantial speedup through learning is vanishingly small. This does not bode well for the possibility to learn a fast universal solver.

To see what happened, it is useful to abstract a bit. Suppose we have a mechanism q(h) generating trajectories. Let $D=\{h_{1},\ldots ,h_{n}\}∼q$ be data seen in the past (our training set), and let $h_{\mathrm{new}}∼q$ be new data we are trying to find at inference times. This forms a graphical model:


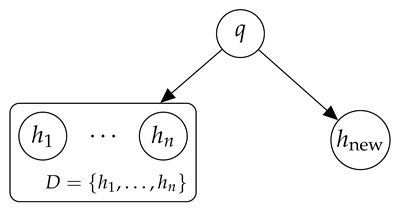

where *q* acts as a separator between past and future data. By the Data Processing Inequality [4], this implies
  (22) $$I\left(D:h_{\mathrm{new}}\right)\leq I\left(D:q\right)\leq K\left(q\right).$$
  That is, since $h_{\mathrm{new}}$ is sampled i.i.d. from *q*, the only information that past data *D* can provide about $h_{\mathrm{new}}$ is information about *q* itself, and this cannot be larger than its description length K(q).

**Theorem 12** 
(Maximum speedup is bound by world complexity)**.** *The maximum speed-up we can obtain using data generated by process q is*

(23) $$\log\frac{\tau_{\nu }\left(h\right)}{\tau_{\nu }\left(h|D\right)}=K\left(q\right)$$

Since the universal prior m(h) has low Kolmogorov complexity K(m)=O(1), there is nothing we can learn from it. (This may be slightly confusing; *m* can generate programs of arbitrary complexity, but *its own complexity* is low. In fact, we just need a few lines to define it). More generally, whenever the data is generated by a low-complexity distribution, no matter how much data we observe, we will never be able to obtain more than a constant time speed-up.

This gets to a key question about what is the scaling law of information for real world data. To study it further, it is useful to reframe the question a bit. We have been thinking of *q* as a mechanism that generates i.i.d. samples of trajectories. This may be restrictive. More generally, let *q* be a dynamical process generating a sequence $x_{1},x_{2},\ldots$ of tokens. Let $X_{n}=x_{1:n}$ and $Y_{m}=x_{n:n+m}$ be an initial sequence of length *n* and its continuation of length *m*. We can think of $X_{n}$ as our training set (past data) and $Y_{m}$ as our test set (future data we are trying to predict). It may be useful to think of $X_{n}$ and $Y_{m}$ as being natural language text or code.

We want to know how $I(X_{n};Y_{m})$ scales when n,m→∞. Let us suppose *q* is a finite-dimensional Markov process with a discrete *D*-dimensional hidden state $s\in S^{D}$ over some alphabet *S*. What information can $X_{n}$ provide about $Y_{m}$? By the Markov hypothesis, the only information that $X_{n}$ can provide to help predict $Y_{m}$ are the parameters θ of the underlying process and the final state $s_{n}$, so we have

(24) $$I(X_{n}:Y_{m})\leq c|\theta |+D\log|S|$$

where |θ| is the number of parameters of the process and *c* is how many bits we need to encode the parameters. Again, we find that for a very large class of processes, $I(X_{n}:Y_{m})$ is bounded by a constant, and, asymptotically, there is nothing to learn as long as (i) the parameters of the process are finite-dimensional and (ii) the size of the state is bounded (or, equivalently, the process has finite or fading memory).

### 4.1. Hilberg’s Law for Scaling

Is this what happens on real data? A particularly well studied case is when the process *q* generating the data is a human writing natural language text. In the special case that n=m, **Hilberg’s law** [12,31,32,33,34,35] posits that

(25) $$I\left(X_{n}:Y_{n}\right)\propto n^{\beta }$$

for some 0<β<1. This is in sharp contrast with the results above. If Hilberg’s law holds (which, empirically, it does [36]), then the process generating real data is very unlike any standard dynamical process. (An unrelated consequence is that a pure LLM implemented by a *state space model* or an attention model with finite context cannot possibly be a perfect model for natural language. Since its state is bounded, it satisfies Equation (24) and cannot asymptotically scale like natural text. However, RAG sidesteps the issue, so an agent with external memory can be a model of language, or a model of the world, in ways in which an ordinary Transformer cannot no matter how many parameters it has and how much data it is trained on).

Since we care about real data, let us introduce the following generalized Hilberg’s law (GHL) scaling to arbitrary *n* and *m* and take it as our assumption of how physically generated data, including human-generated data, behave.

**Definition 5** 
(Generalized Hilberg’s law)**.** *Let $X_{n}=x_{1:n}$ and $Y_{m}=x_{n:n+m}$ be data generated by a stochastic process. We say that it has GHL scaling if*

(26) $$I\left(X_{n}:Y_{m}\right)\propto n^{\beta }+m^{\beta }-{\left(m+n\right)}^{\beta }$$

This expression reduces to the standard Hilberg’s law when n=m. It is symmetric and always positive. (Define s=n/(m+n) and t=m/(n+m). The function $f\left(x\right)=x^{\beta }$ is convex, so $s^{\beta }+t^{\beta }\geq {\left(s+t\right)}^{\beta }=1$). To get an intuition of how a process may satisfy the GHL, in Section 4.4, we will show one explicitly based on the *Santa Fe process* [34,35]. The key intuition will be that the GHL is satisfied whenever the “world” (whatever is generating the data) contains an unbounded amount of unchanging *facts* that are referenced in the data with a heavy tail distribution. For now, let us assume that our process satisfies the conjecture and derive the scaling laws for speed-up of a universal search agent.

### 4.2. Scaling Laws for Time

Assume the training data $X_{n}$ and the inference data $Y_{m}$ are generated by a process satisfying the GHL:

(27) $$I\left(X_{n};Y_{m}\right)=m^{\beta }+n^{\beta }-{\left(n+m\right)}^{\beta }.$$

We are interested in the case where $X_{n}$ is the training set, so n≫m, in which case, we can approximate

(28) $$I\left(X_{n};Y_{m}\right)\approx m^{\beta }-\beta \frac{m}{n^{1-\beta }}\;.$$

From Theorem 11, the log-speed-up we get from training is exactly $I(X_{n};Y_{m})$ and m=T(h) is the length of the inference time trajectory. Therefore, we conclude as below.

**Theorem 13** 
(Time-Scaling Law)**.** *The log-speed up we obtain training on a large enough dataset D of n tokens is*

(29) $$\log\frac{\tau_{\nu }\left(h\right)}{\tau_{\nu }\left(h|D\right)}=T{\left(h\right)}^{\beta }-\beta T\left(h\right)/n^{1-\beta }$$

This tells us a few interesting things. First, the speed-up is upper-bounded not by a constant (like we previously obtained for simple models) but by $T{\left(h\right)}^{\beta }$. That is, the longer the trajectory is, the more it is sped up by learning. This makes intuitive sense: if finding a solution required just a few steps, even without any learning we could have brute-forced it quickly. Complex problems are the ones that benefit the most from learning. We also get $O(T\left(h\right)/n^{1-\beta })$ convergence to the optimal speed-up, so we want the number of training tokens *n* to be

(30) $$n\propto L^{1/(1-\beta )}.$$

where *L* is the maximum length of the trajectory we expect to need to solve a problem. That is, we need more training tokens if we expect to solve challenging problems.

The parameter 0<β<1 relates to the *complexity* of the task distribution; in particular, it controls how long-tailed the distribution of ‘useful facts’ is, with β→1 implying that the distribution is very heavy-tailed. When β is high, we need significantly more training tokens to achieve the optimal rate since there are many more facts that are commonly used. But we also get a better payback since the speed-up $T{\left(h\right)}^{\beta }$ is also going to be larger.

**Remark 3.** 
*For natural language, β≈0.8 [36], which gives $n\propto L^{1.25}$. So, if we are going to generate trajectories of 10K tokens, we need ≈100K training tokens.*

This ratio of test to train data is realistic when fine-tuning a model for reasoning. But when training from scratch, it is a clear underestimate. There are a few factors to consider. First, the initial training data is needed to put the weights in a proper configuration, which depends more on the amount of weights than on the information in the training data. Indeed, it is common to pretrain on lower-information content with size proportional to the number of weights. Second, we are assuming that the mechanism generating the test data is the same as the training data, which is not the case. Facts that are useful at test time may appear very rarely in the training set (e.g., if we ask PhD-level questions to a model trained on generic data). Third, the scaling laws are derived under the assumption that we can identify useful facts and memorize them the first time we see them. But, realistically, we need to see a fact multiple times to identify it as useful, which inflates the number of required tokens.

### 4.3. Memory-Time Trade-Off

So far we have assumed that we can use all information in *X*, but, in practice, the available memory *M* may be a bottleneck. On a dataset of length *n* there are $k=n^{\beta }$ facts to memorize, requiring M=ck bits of memory. Replacing *n* with *M* using this relationship in Theorem 13, we get the scaling if memory (rather than *n*) is the bottleneck.

**Corollary 2** 
(Time–Memory Scaling Law)**.** *Assuming memory is used optimally, the speed-up as a function of the used memory is given by*

(31) $$\log\frac{\tau_{\nu }\left(h\right)}{\tau_{\nu }\left(h|D\right)}=T{\left(h\right)}^{\beta }-\frac{T(h)}{M^{1/\beta -1}}$$

However, this assumes that we are somehow able to extract from the training data the most useful facts and store them (and only them) in memory. Since we are using an online learning algorithm, the memory also needs to store information about the facts in the training data that we have not yet deemed useful, since we need to wait to see them again to confirm if they are useful.

**Proposition 1** 
(Online Memory Overhead)**.** *An online agent needs a constant factor*

(32) $$M_{\mathrm{online}}=C_{\beta }M_{\mathrm{offline}}$$

*of additional memory compared to offline to achieve the same performance.*

This reflects a realistic issue: it is easier (i.e., faster) to learn from a textbook that gives us directly the useful facts (offline learning) rather than having to ‘connect the dots’ and try to guess the useful facts from online experience.

#### Prompting and RAG

So far we have focused on the speed-up provided by training on dataset *D* of past data. What is instead the effect of adding prompt *p* to the request? First, note that the key result,

(33) $$\mathrm{speed}\text{-}\mathrm{up}=2^{I_{\nu }\left(h:p\right)}$$

where dataset *D* is replaced by prompt *p*, remains valid, so the speed-up is still determined by the ν-algorithmic mutual information between the prompt and the trajectory.

If the prompt is an *in-context learning* prompt, which provides examples of the task, then the theory is identical to the case of a dataset (effectively, the prompt *is* a dataset). However, we expect it to provide much more algorithmic information per-sample than pre-training dataset *D* since presumably it will contain only examples directly relevant to the task.

The prompt could also contain information directly relevant to the trajectory, which does not follow a GHL scaling law. For example, if the prompt is a *plan* describing exactly what to do, then

(34) $$I_{\nu }\left(h:p\right)=\ell_{\nu }\left(h\right)-\overset{0}{\overset{︷}{\ell_{\nu }\left(h|p\right)}}=\ell_{\nu }\left(h\right)$$

and we get the maximum possible speed-up, meaning that the time to execute the search becomes merely $\tau_{U}=T\left(h^{*}\right)$, the minimum possible time required by a trajectory to solve the task.

Alternatively, a prompt *p* may not specify the whole trajectory, but all the information that it has may be relevant to the trajectory; that is, $I\left(h:p\right)=\ell_{\nu }\left(p\right)$. In this case, we get a significant speed-up $2^{\ell_{\nu }\left(p\right)}$. For example, just 10 good bits of prompt (a few tokens) can reduce the time to find a solution by ∼1024 times. We can think of this as a useful *hint* (“*Solve the following task. The following technique may be useful: …*”) that brings down the time to solve a problem from hours to minutes.

### 4.4. Example of GHL Scaling: Santa Fe Process

So far we have assumed that our data generating process satisfies the Generalized Hilberg’s law scaling $I\left(X_{n}:Y_{m}\right)=n^{\beta }+m^{\beta }-{\left(n+m\right)}^{\beta }$, and we anticipate that this relates to having an infinite distribution of facts that appear in the data following a long-tailed distribution. Following [31], we now explicitly construct such a process, showing that the GHL scaling definition makes sense and how exactly ‘facts’ relate to scaling.

Let ${\left\{Z_{k}\right\}}_{k=1}^{\infty }∼\mathrm{Bern}\left(1/2\right)$ be an infinite set of binary properties that are sampled *before* any text is generated. We can think of them as facts about the world, which may be referenced in the text. Importantly, since the $Z_{k}$ are sampled only once at the very beginning and do not change over time, once a fact is first encountered, we know its value in any future text. (An alternative view is that the process has extremely long memory: after the first time it generates a value for $Z_{k}$, it remembers it and reuses at all later times). Some facts are referenced very often, others very rarely. Empirically, natural frequencies are well captured by a Zipf power law:

(35) $$p\left(k\right)=ck^{-1/\beta }$$

for some normalization factor *c*.

To generate a sequence *X*, we concatenate the index of a random fact and its value:

$$X=(\left(k_{1},Z_{k_{1}}\right),\left(k_{2},Z_{k_{2}}\right),\ldots ,\left(k_{n},Z_{k_{n}}\right))$$

where $k_{i}∼p\left(k\right)$. We generate *Y* similarly.

**Theorem 14** 
(Santa Fe Process GHL Scaling)**.** *The Santa Fe process described above follows GHL scaling:*

$$I\left(X_{n}:Y_{m}\right)=n^{\beta }+m^{\beta }-{\left(n+m\right)}^{\beta }$$

**Proof.**  We now want to show that for this process, $I(X_{n};Y_{m})$ follows GHL scaling. Let us first rewrite

I(X:Y)=H(X)+H(Y)−H(XY).

To compute the compression cost H(X) of *X*, the key observation is that we only need to encode the value of a fact the first time we see it (since it remains constant). How many unique facts appear in *X*? Asymptotically, this is given by

$$U\left(n\right)=\sum_{k=1}^{\infty }\left[1-{\left(1-p_{k}\right)}^{n}\right]\approx C_{\beta }n^{\beta }.$$

Using this, the compression cost H(X) is the cost of encoding the *n* random indices (using H(p) bits per index), plus the cost of encoding the U(X) unique properties:

$$H\left(X\right)=nH\left(p\right)+C_{\beta }n^{\beta }.$$

The cost of H(Y) and H(XY) are computed similarly. Putting all together, we get the desired result:

$$\begin{array}{cc} I(X;Y) & =H(X)+H(Y)-H(XY)\\ \; & =\left[nH\left(p\right)+C_{\beta }n^{\beta }\right]+\left[mH\left(p\right)+C_{\beta }m^{\beta }\right]-\left[\left(n+m\right)H\left(p\right)+C_{\beta }{\left(n+m\right)}^{\beta }\right]\\ \; & =C_{\beta }\left[n^{\beta }+m^{\beta }-{\left(n+m\right)}^{\beta }\right] \end{array}$$

□

This construction is quite artificial—real world data are not a stream of random facts, indices, and immutable binary values. But it does highlight in a simple way some key, and more fundamental, issues: First, the process has *infinite complexity* since it needs to store the value of all the $Z_{k}$. Recall that by Theorem 12, this is exactly the setting where learning can make the most difference, since we can obtain arbitrarily large speed-ups. Second, the distribution of facts has to follow a power law. Some facts (“*the color of the sky*”) are more referenced than others (“*the house number of John Doe*”) and power laws are abundant in real data and have several theoretical justifications (rich-get-richer effect [37], least-effort/max-entropy trade-off [38], monkey-typing with intermittent silences [39,40], etc.).

In terms of reasoning traces, one may think of ‘facts’ as *functions* or *theorems* that one may invoke by calling their name (index). Since functions/theorems are reused and always remain constant; after memorizing them, we can significantly compress future reasoning.

This intuition has motivated a significant volume of research regarding universal solvers [3,41,42,43], program synthesis [44,45,46,47], and reinforcement learning [48,49].

## 5. Inversion of Scaling Laws

So far we have established the fact that learning from data leads to a speed-up in finding a solution to an unforeseen task. However, the equivalence

(36) $$\log\left(\mathrm{speed}\text{-}\mathrm{up}\right)=I_{\nu }\left(h:D\right)$$

tells us something stronger: we learn to be faster *if and only if* we learn from data. This suggests that we can learn something from data if and only if we train with a time optimization objective.

Let us work through an example. Suppose we want to train a universal solver, and (as is natural) we use as reward function the expected number of correct solutions, determined by a function *R*, averaged over some distribution f∼q of tasks:

(37) $$L=E_{f∼q}E_{h∼\nu }\left[R\left(h\right)\right].$$

Further suppose that our agent has unlimited computing power available, so that we have no need to optimize resources over their usage. What will such a system learn?

If the distributions of tasks is generic enough, we know that the system has to learn to perform universal computation (Theorem 7). But that is *the only* thing that it needs to learn. Having universal computation, it can implement the basic Solomonoff–Levin universal search algorithm, which will always find the solution to the task, thus achieving maximum reward. It will take eons to find the solution, but since computing is free for this agent, that is not a problem.

To further clarify, suppose we want to teach the model to play chess. Training is not necessary to achieve a better reward, since a standard tree-search over all the possible moves will eventually find the best move to make. Training is required only to *reduce the time* that it takes to find the best move.

**Remark 4.** 
*Only time bound systems learn. If a system is not penalized for the time it takes to find a solution to the task, it is optimal to always brute-force a solution without learning anything. Vice versa, any system that optimizes time has to learn at least $I_{\nu }\left(h:D\right)=\log\left(\mathrm{speed}\text{-}\mathrm{up}\right)$ bits of information from the data.*

**Figure 1:**
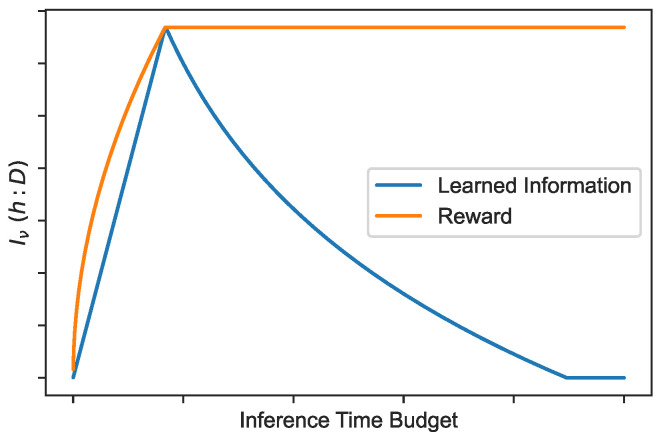
Simplified illustration of expected scaling law inversion curve. Consider wanting to solve a task—initially requiring time *L*—in less than *T* reasoning steps. Further assume we model capacity (number of weights) with the time budget *T*. Initially, the model stores as much information as its weights allow to reduce the required time from *L* to less than *T*. Once $I\left(h:D\right)=\log\frac{L}{T}$ bits are stored, the task is solved exactly (the reward is maximized). Beyond this point, increasing *T* may actually reduce stored information, since less is needed to meet the time budget. At T=L, no information needs to be stored to achieve perfect reward.

Going more in depth, we can look at how we expect $I_{\nu }\left(h;D\right)$ to behave as we scale the model. First, note that as we scale the maximum time allowed for a trajectory, we also usually want to jointly scale the amount of weights of the model [10]. So, if *T* is the maximum time for the trajectory, the number of weights will be some monotone function |θ|=f(M). Note that the number of weights puts a constraint on the maximum amount of information I(ν:D) about the data that we can store in the model parameters. But since $I_{\nu }\left(h:D\right)\leq I\left(\nu :D\right)$, this also puts an upper-bound on the per-trajectory information $I_{\nu }\left(h:D\right)$. Next, let us look at how much information the model is forced to capture if it wants to have perfect performance on the task. We need $\tau_{\nu }\left(h|D\right)\leq T$, so we need to store enough information to speed up the search until it takes less than *T* total time. This means that

(38) $$\begin{array}{cc} \tau_{\nu }\left(h|D\right) & =\frac{\tau_{\nu }\left(h\right)}{2^{I(h:D)}}\leq T \end{array}$$

(39) $$\begin{array}{cc} \Rightarrow I(h:D) & \leq \log\tau_{\nu }\left(h\right)-\log T \end{array}$$

so as expected from the discussion before, the amount of information we *need* decreases as *T* increases. Putting the two bounds together, we obtain the curve for $I_{\nu }\left(h:D\right)$, shown in Figure 1.

As we scale the model, when the inference time budget and the number of weights are small, the model learns as expected, acquiring information from the data, storing it into the weights. The expected reward it obtains steadily increases as more problems become solvable within the allotted time budget. At some point, the amount of information the weights can store is large enough that, thanks to the speed up, all trajectories are solvable within the time budget. At that point the reward is always optimal and stabilizes. But, paradoxically, if we further increase the time budget, the model can use brute force search more, and the information it needs to acquire starts *decreasing* until it reaches zero. This is the *savant regime*, where the model can default to expensive brute force search with no learning whatsoever, yet still achieve the optimal reward. In this regime, optimal performance (orange curve) comes from excess capacity rather than “insight,” as measured by learned algorithmic information (blue curve).

To avoid entering the savant regime, one option is to penalize time using a reward like

(40) $$\begin{array}{cc} R & =R\left(x,y\right)-\lambda \log\tau_{\nu }\left(x\mapsto y\right) \end{array}$$

(41) $$\begin{array}{cc} \; & =R\left(x,y\right)+\lambda I_{\nu }\left(h:D\right) \end{array}$$

This forces the model to actually learn algorithmic information. The training of many current large reasoning models [50,51] includes similar objectives that maximize reward while minimizing inference time. The results above suggest that this regularization not only reduces inference cost—a common motivation for penalizing time—but also improves the quality of what the model learns.

Note the sharp contrast with information theoretic regularizers. In a classic machine learning setting, we want to maximize [52]

(42) $$R_{\mathrm{reg}}=R\left(x,y\right)-\alpha I\left(w:D\right)$$

That is, we want to **minimize** the amount of information in the weights [6]. This ensures generalization, and is the basis of the Minimum Description Length (MDL) principle. For reasoning, however, we want to **maximize** the information in the weights in order to minimize time. In transduction in a verifiable setting, there is no issue of generalization, since we have access to all relevant data, and a verifier can that tells us whether the task has been solved. More generally, the trade-off is not trivial.

Consider a real biological agent that has to model a physical environment in order to act. The agent may learn the correct, generalizable, underlying physical rules that would be optimal from an MDL perspective. But if those rules involve a large amount of computations, the agent may not be able to use them in a reasonable time for survival. It may instead be optimal for the agent to memorize several case-by-case rules (e.g., a feather falls with a certain speed) even if they do not generalize (a feather does not fall with the same speed in a vacuum), if they allow it to quickly come up with approximate, but timely, estimates that are better for survival than the best estimate rendered belatedly.

It is common to refer to transduction as *System-2* behavior and induction (restricted to a single forward pass) as *System-1* behavior [53]. Encoding reasoning traces into the weights can then be thought as *automatization*: the common thinking patterns are made faster by moving them to *System-1*. Whether this is advantageous depends on environmental stability. In stationary environments, automatization allows faster reaction and better energy usage. However, in time-varying environments, the ability to reason at inference time cannot be fully replaced by a set of fixed learned behaviors.

## 6. Maximizing a Continuous Reward

So far we have studied universal solvers under the assumption that the reward function R(x,y) has a binary “success/failure” value. In the more general case, the universal solver is required to maximize a continuous reward function. If we know the maximum achievable reward $R_{\max}$, and we know that the reward can be achieved, we can define a new binary reward function “has achieved the maximum,” thus reducing to the binary case. However, generally, we do not know what is the achievable maximum and, more importantly, we do not know if the search cost to find a better solution will be worth the value. That is, *a practical universal solver has to decide when to stop*.

The objective underlying this decision is optimization of the user’s net gain. Consider a universal solver *U* that over time outputs various candidate solutions $y_{t}$, and let $R^{*}=\max R\left(x,y_{t}\right)$ be the maximum reward achieved by any solution up to now. The value obtained by the user stopping at this point is

$$J=R^{*}-\lambda T_{\mathrm{total}}$$

where $R^{*}$ represents the best reward achieved across all explored traces, λ is the per-token cost, and $T_{\mathrm{total}}$ is the total compute time consumed until now. The question is whether investing additional compute to search for a solution is likely to improve *J*.

Deciding whether a reward function can be improved, let alone whether it is convenient to do so, is generally undecidable. Hence, we need to assume we have learned a *forecasting model* $\psi_{\theta }$ to estimate the distribution of rewards for each potential continuation *h*, enabling principled decisions about which reasoning paths to pursue [18]. Under this assumption, we can formulate the search for optimal solutions as a *Pandora’s box problem with order constraints* [19,20].

**Universal search as Pandora’s box problem.** We can visualize all possible computations to solve a task as a rooted tree T. A node *n* represents a partial reasoning trace; exploring an extension of a reasoning trace (i.e., a child of *n*) for *t* tokens incurs a cost c=λt and, if we terminate after the extension, yields a terminal reward $R∼\psi_{\theta }\left(\cdot |x,z\right)$. Nodes naturally obey order constraints: a child may be explored only after its parent.

Given the current best obtained reward $R^{*}$, the *incremental value* of exploring a new child node *n* is

(43) $$\Delta \left(n\right)\;=\;E\;\left[{\left(R-R^{★}\right)}_{+}\right]\;-\;\lambda t.$$

While we could greedily pick the next computation to perform by maximizing Δ, this generally leads to suboptimal solutions. Searching for the best possible strategy is, in principle, exponentially complex. However, Weitzman [19] showed that a simple greedy strategy does exist to optimally solve this problem. In particular, Weitzman defines a *conditional reservation value* for each candidate extension as the unique $z_{n}$ solving

(44) $$E\;\left[{\left(R-z_{n}\right)}_{+}\right]\;=\;\lambda t.$$

The resulting value of $z_{n}$ is called the *Gittins index* of the node. The provably optimal policy is then to visit at each step the unexplored node that has the currently highest Gittins index. It is instead optimal to *terminate* the search when

(45) $$R^{★}\;\geq \;\max_{n\in \mathrm{unexplored}}z_{n}$$

at which point there is no node that we can visit that is expected to improve the final objective *J*. That is, the expected improvement in reward does not compensate for the cost of exploring the node.

This framework can be extended [20] to the case when there are constraints on the order of opening the boxes (e.g., testing a solution obtained after 1024 thinking tokens requires first reaching 512 tokens), in which case the value of a box also needs to take into account the value of the boxes it allows access to.

**Making decisions with Gittins Indexes.** Once we use the Gittins index $z_{i}$, using our forecasting model, we have a simple criterion to make several key decisions during our search. For example, $z_{i}$ allows us to decide: (i) when to *continue extending a reasoning trace* (if the Gittins index of a child is the highest); (ii) when to branch a trace (if the child of a parent node achieves the highest index), in particular when it is optimal to restart from scratch by sampling a new reasoning trace); and (iii) when it is optimal to stop attempting to improve the solution of a problem and return the current best (when no node has a better index than the current reward).

**Need for a Forecasting Model.** Exploring greedily based on Gittins indexes remains optimal as long as: (i) the reward distribution is known in advance and (ii) the distribution of different boxes are independent (the reward observed for one box does not affect the predicted reward on others). When this is not the case, using Gittins indices may not be optimal, but generally remains a strong policy [54].

In general, however, we do not know *a priori* the distribution of possible rewards that we may obtain. For example, we cannot know in advance that thinking for 1000 or 10,000 tokens will lead to finding a correct solution. In order to efficiently optimize the net reward *J*, an agent also has to learn to *forecast* both the cost of an exploration attempt and the probability of it improving over the current best solution [18].

## 7. Discussion and Related Prior Work

The subject matter of this paper falls within the scope of statistical machine learning. Traditionally, one starts with instantiated data that implicitly define *the task* and arrives at a model that performs the same inference computation on all future instances of the same task. However, once trained models are used as generative distributions, as is customary in generative AI, they exhibit behaviors that were not explicitly encoded in the training data nor the chosen loss. A key such behavior is the ability to perform variable-length inference computation, which increasingly often leads to solving previously unseen tasks. In classical (inductive) machine learning, there is no feedback mechanism at inference time, so one can only evaluate the quality of a model *post-hoc*, typically on data other than the data at hand. Agents, on the other hand, interact with the environment, which provides feedback, and/or can call tools to solicit feedback or spawn simulations at inference times prior to rendering an outcome. Inference computation can therefore adapt depending on the resulting feedback. This mode of interaction calls for a different approach to learning, which aims to empower *transductive inference*. The power of LLMs stems from the fact that, despite being trained *inductively* (despite the name, so-called unsupervised pre-training is simply next-token prediction, a standard multi-class supervised classification problem), they operate transductively, leveraging their chain-of-thought. In this paper, we explore the foundational principles of such *transductive learning*, and its limits, including bounds and power laws.

**Transduction, In-context Learning, and Solomonoff Inference.** Transduction in the form of learning jointly from labeled training examples and unlabeled test samples was championed by Vapnik [1,55,56].

The dichotomy we present between transductive and inductive inference has long been considered in machine learning under various guises, such as the distinction between “learning to generalize” and “learning to reason”, between System 1 and System 2 [53,57], between *fluid intelligence* and *crystallized intelligence* [58], or between learners and solvers [57]. Our objective is not to further justify the need for transduction or reasoning, which has amply been done, but rather to contextualize it through the lens of LLMs as computational engines and provide scaling laws connecting time-to-solve with the learning of algorithmic information.

An early observation was that language models can learn multiple tasks implicitly through the unsupervised language modelling objective [59] and exhibit diverse behaviors when adequately prompted. In-context learning [60], which is a form of transductive inference, introduces demonstration examples into the model context to elicit desired behavior. It has been shown that LLMs can perform optimization algorithms such as gradient descent and and ridge regression transductively at inference time from in-context examples [61]. Ref. [62] demonstrates that sparse linear functions, decision trees, and two-layer networks can be learned in-context. Ref. [63] investigates what minimal pretraining is necessary to induce in-context learning, showing that a small pretrained model can achieve close to the Bayes optimal algorithm. Ref. [64] introduces a theory of in-context learning where a hypothesis is formed at inference time, obtaining generalization bounds. Ref. [65] demonstrates that a single self-attention layer trained by gradient flow to perform in-context learning converges to a global minimum that is competitive with the best predictor on the test distribution. The connection between in-context learning and Solomonoff inference was identified in [66], where the authors attempted to learn the Solomonoff semimeasure directly by sampling programs and training on their outputs. In [67], motivated by the inductive theory of Solomonoff [7,8,68], the authors demonstrate that LLMs can outperform general purpose compressors, even for audio and visual data. The connection between Solomonoff induction and neural network optimization as a form of program search was mentioned earlier in [69].

As we noted in the introduction, time plays no role in Solomonoff inference, nor in in-context learning. Neither involve actual “learning” in the classical inductive sense: The weights are fixed and the same task, presented in-context multiple times, requires repeating the same effort to yield no different outcome each time. However, time plays a key role in *learning transduction*, which is the core motivation of this work.

**Systematicity of Dichotomies.** Several conceptual pairs recur throughout this work (Table 1), which can, among other interpretations, be seen as projections of the distinction between inductive and transductive inference onto different aspects of a learning system.

In inductive inference, the goal is to compress past observations into a fixed map that generalizes to similar future inputs. The bottleneck is representational capacity (space/parameters); success is measured by accuracy on held-out data; simplicity aids generalization; and memorization of training-specific detail is harmful. Computation is a single feedforward pass (System 1), interpolating among previously seen patterns. Space-pressure (amount of information stored) is important to learn generalizable features rather than memorizing past results. A prototypical task is language modeling: reducing uncertainty on the next token given the statistical structure of past text.

In transductive inference, the goal is to reason about a *specific* instance at inference time, guided by a verifier or reward signal. The bottleneck is computational budget (time/tokens); success is measured by speed to a verified solution; and memorization of reusable algorithmic structure is beneficial (Theorem 11). Computation is variable-length chain-of-thought (System 2), extrapolating beyond the training distribution to solve novel tasks. Time pressure is essential for learning to occur (Theorem 4) and avoid savantry. A prototypical task is theorem proving: search for a proof that satisfies a checker.

The two regimes are not mutually exclusive. Automatization (Appendix A) moves frequent reasoning patterns from the transductive to the inductive regime, trading space for time. Conversely, when the environment changes, previously automatized behaviors must be re-derived transductively. A complete agent must balance both, and the optimal operating point depends on the stability of the environment and the cost of computing.

**LRMs, SLMs, VLMs, VLAs, World Models, etc. (nomenclature).** The term LLM originally referred to large-scale Transformer-based models (pre-)trained as next-token predictors using large-scale corpora of natural language, then co-opted as probability distributions to sample new natural language text. Optionally, these models could be fine-tuned by scoring such generated expressions using human preference, an external reward mechanism, or the model itself through self-assessment. It is also common to call the outcome of the same process a ‘World Model’ (WM) if trained on sensory data such as video or audio instead of natural language, a ‘vision-language model’ (VLM) if trained on both, or a ‘vision-language-action’ model if the output expression is used to issue commands to an actuator, or ‘large reasoning models’ (LRMs) if they are used to generate variable-length trajectories prior to rendering the decision or action. In our nomenclature, any large-scale predictor trained on sequential data with latent logical/linguistic structure (with objects, relations, functions, etc.) develops an inner state space with an internal “Neuralese language” [70]. Sensory data are replete with latent discrete entities [71], their relations (topological, geometric, dynamic, semantic), and (de)composition into parts (meronomies) or abstract concepts (taxonomies). In our definition of LLM, therefore, where ‘language’ is not restricted to natural language and instead refers to any form of Neuralese, VLMs, WMs, VLAs, LRMs, and other variants are also LLMs. We also include in the term LLM models that use different architectures, so long as they have a ‘state’ (memory), whether explicit (as in state-space models) or implicit by co-opting a sliding window of data, as in autoregressive Transformers [72]. Since the largest LLMs at this point comprise trillions of parameters, some now refer to models with merely billions of parameters as ‘small language models’ or SLMs. Obviously, ‘small’ is subjective, and these models have no architectural, structural, functional, or conceptual difference from their ‘large’ counterparts, so they too are just LLMs. Empirically, some emergent phenomena are only observed at scale, but this does not mean that there is a clear divider between ‘large’ and ‘small’, even phenomenologically since smaller models can still be distilled from larger ones and maintain their behavior even if it would not have emerged from cold-start using the same training protocol [50].

**Embodied AI.** The results described in this paper pertain to both software agents that exist within the world of bits and embodied agents that interact with the physical environment. While this may seem counter to the forced dichotomy between LLMs and so-called World Models, once the sensory data is tokenized, the two become the same mathematically. Regardless of how a model is trained inductively, once it acquires the ability to perform transductive inference, it can act as an agent. This could be in the world of bits, where interaction with the surrounding environment is through APIs and function calls, or in the world of atoms, where sensors provide measurements that the agent turns into a representation of the environment (which is an abstract concept finitely encoded in the state of the agent [73]) and operate on it (i.e., reason) to command physical actuators that affect the the environment. Such an environment then provides a feedback signal, ultimately in the form of “verification” (e.g., survival or rewards). The reasoning agents exist in a finitely encoded world and interface with the physical world through encoders and decoders. The core of all these agents is the ability to perform transductive inference within the discrete/discretized representation, which requires computation as described in this document. While evolution proves that processing sensory data is sufficient to foster the emergence of reasoning, language is already conveniently distilled (symbolized and compressed), making the traversal of the evolutionary path unnecessary for the emergence of reasoning. In this sense, agentic AI subsumes embodied AI, where the latter focuses on the source of the data (sampled physical sensory measurements) and focuses on physical actuators commands (physical action).

**Universal Computation, Universal Search, Algorithmic Information.** Theoretical Computer Science has devoted decades to the development of universal algorithms; indeed, Levin’s paper that introduced his universal search algorithm seeded a large portion of the subsequent literature on computational complexity theory. Since we only use the concepts driving universal search, we do not review this sizable body of work here and refer the reader to any textbook on complexity theory. One exception we make is to comment on the literature of Kolmogorov Complexity and Algorithmic Information Theory, which is thoroughly covered in textbooks [5].

While Kolmogorov’s theory is useful for certain asymptotic analysis, and we make heavy use of it in this manuscript, it is worth pointing out that it fails its original intent to canonically separate “information” from “randomness’ with the concept of the Structure Function. In our approach, the information in a trained model is specific to the model and, hence, subjective.

**Reinforcement Learning.** Our formulation—searching for $y^{*}\left(x\right)=\arg\max_{y}f\left(x,y\right)$ given a score function *f*—superficially resembles reinforcement learning (RL), where an agent maximizes cumulative reward through interaction with an environment. However, the goals differ in a key way. Standard RL seeks to learn a fixed *policy* for a *particular* environment. We instead want a universal solver to craft a *new* policy for tasks no agent has previously encountered. Put differently, a particular RL problem is one possible task *f* that our universal solver could be asked to address, but the solver itself is not specialized to any single such task. While RL considers time-penalized objectives, the time RL tries to minimize is the number of actions or observations, while we try to minimize the computing time used by the agent to *decide* which actions to take or to process the observation. The theoretical questions we focus on—how learning compresses the multiplicative constant $2^{\ell (A)}$ of universal search and how scaling laws govern the resulting speed-up—are orthogonal to the design of any particular RL algorithm, which is why we do not review that extensive literature here. As an anonymous reviewer pointed out, the term “agent” has traditionally referred to algorithms whose every action affects the state of the world, whereas algorithms that perform computation without affecting the world ought to be called “reasoners.” However, modern AI agents can, and do, perform actions such as retrieving data, executing code, or running a verifier that do not change the state of the world (thus fitting within the search paradigm), yet nonetheless represent actions triggered by the chain of thought, which justifies the customary use of the term “agent.”

**Markov Chains.** We use general dynamical systems as a model of computation, but arguably the most important piece is the transition probability $\nu (s_{t+1}|s_{t})$, which defines a Markov Chain. One may wonder why we need to introduce such general machinery, including ‘proper time,’ when standard concepts from Markov Chains, such as the expected hitting time, would suffice. As noted, however, hitting time could be made arbitrarily small or large without fundamentally changing the computations performed. We also note that since AI agents interact with the unknown environment, they are not closed systems describable with a Markov chain, but can still be described as a dynamical system. We also note that our use of dynamical systems or Markov structure is restricted to modeling the *computations of the agent* solving the task, not on the data itself. In fact, we stay clear of ever making any assumption on the data generating distribution aside from it satisfying the Hilberg’s law, which defies the Markov assumption. We also note that, while ultimately one could argue that any physical process has a latent finite-memory or Markov structure, there is a fundamental difference between a Markov process of a *known* order and one of a finite but unknown order. In the former case, one can just instantiate a model with sufficient capacity and know that it will learn the statistics of all order. In the latter case, one can never know when they have observed enough, and must instead be ready to add to memory—effectively treating the process as non-Markov. The theory described in this manuscript pertains to the latter case.

**Memorization and Generalization.** Information complexity-based generalization theory formalizes the notion that generalization occurs whenever the information the learned hypothesis contains about the training data is minimal (low memorization). Ref. [74] demonstrated that a single information exponential inequality is sufficient to derive PAC Bayes bounds [9], the mutual information bound [75], and a version of the conditional mutual information bound [76]. Even the classical finite-hypothesis and VC dimension bound [77] can be viewed as primitive versions of such bounds. All the aforementioned results assume that the training and test data are drawn as i.i.d. samples from a common distribution. In contrast with the aforementioned theory, it has been demonstrated that there are learning tasks where memorization is provably necessary to achieve high accuracy [78], and that mitigating memorization can cause the model to fail on long-tailed tasks [79,80]. There is evidence that natural language is akin to such long-tailed tasks that require memorization. Recent work demonstrates that there are models for language that explicitly memorize the training data that perform well. Ref. [81] introduces nearest-neighbor language models (kNN-LM) that predict the next token according to the *k* nearest neighbors of the context embedding, which requires explicitly encoding all context embeddings and their subsequent tokens. Ref. [82] demonstrates that augmenting parameteric LLMs with a kNN-LM can significantly boost performance. Even stronger, ref. [83] demonstrates that a generalization of an *n*-gram model (dubbed ∞−gram) outperforms kNN-LM, while losslessly encoding the training data into a suffix array data structure.

**Additional Related Work.** Ref. [84] championed the analysis of inductive learning from the lens of algorithmic complexity. Ref. [85] provides a comprehensive view of the compression approach to learning algorithmic information, complementary to ours.

Ref. [86] develops reinforcement learning algorithms with insights from algorithmic information theory. Ref. [87] uses Levin search to find *low-complexity solutions* to a classification task, whereas we use Levin search to define a notion of time for universal task solvers. Ref. [41] notes that solutions generally share zero algorithmic information and highlights the importance of learning for decreasing time. They also use an adaptive Levin search with information from the past, although the final solution bears no similarity to our approach. Ref. [42] applies ideas from active learning to general problem solving.

Finally, the study of dynamical systems as a universal computer was developed extensively in the context of hybrid (continuous/discrete) systems, as exemplified in [88]. Although the type of dynamical systems we describe here evolve in discrete space and time, some of the themes are recurrent and additional insights may be gleaned from revisiting that literature.

## Figures and Tables

**Table 1:** Dichotomies outlined in this work can be viewed as projections of the induction–transduction distinction. Each row contrasts the same system property under the two regimes.

Mode of Inference	Inductive	Transductive
**Bottleneck**	Space (parameters)	Time (tokens/computing)
**Transferability**	Interpolation	Extrapolation
**Memorization**	Harmful (overfitting)	Beneficial (speed-up)
**Inference mechanism**	System 1 (fixed pass)	System 2 (variable reasoning)
**Sample task**	Language modeling	Theorem proving
**Scaling**	Accuracy vs. size	Speed vs. size
**Information objective**	Minimize I(w:D)	Maximize I(h:D)

## Data Availability

The original contributions presented in this study are included in the article. Further inquiries can be directed to the corresponding authors.

## Appendix A. Maximalistic Models of Computation

The scaling law for speed discussed in Section 4 describes the gains obtainable by assigning higher probability to common patterns learned from past reasoning traces. However, this is not yet the maximum speed-up we can achieve. Consider, for example, a universal task solver that is frequently asked to solve tasks requiring the computation of a Fast-Fourier Transform (FFT). Once it learns from past data the correct algorithm to compute an FFT, it can instantiate that procedure each time, significantly reducing exploration cost. However, it still must execute hundreds of algorithmic steps—thousands of “thinking tokens” for an LLM—to reach a solution. This is far from optimal: a better solution would be to compute the FFT in a single step, either by modifying the weights to implement the relevant circuitry or by invoking a specialized tool.

Two forces drive such further speed gains: *automatization* and *abstraction*. In automatization, as we have discussed, skills migrate from costly System-2 processes to fast System-1 routines. Instead of re-deriving physical laws, the model learns stable approximations of relevant dynamics; instead of re-searching for an FFT, it executes a compact surrogate. In abstraction, long chains of reasoning are compressed into atomic operations. For example, rather than re-proving a property whenever needed, the model introduces a lemma: an invocable unit that replaces a lengthy sub-derivation.

Both phenomena can be understood as expansions of the model’s effective instruction set. In a CPU, each executed instruction modifies the internal state, and a carefully constructed minimal instruction set (RISC) (Reduced Instruction-Set Computer) suffices for universality. However, some long patterns of operations may appear frequently. This motivates the introduction of complex instructions (CISC) that, even if technically redundant, can perform the same long state transition in a single operation, trading additional real estate (e.g., area) for reduced latency. That is, space redundancy is used to buy time minimality. Similarly, each generated token in a reasoning LLM updates its internal state. The system may be universal even with simple states and transitions. Yet, instead of emitting hundreds of primitive tokens, we can introduce specialized tokens/dynamics—or dedicated callable tools—that let the model ‘jump’ directly to the same final state without having to run through the individual steps.

This introduces a familiar trade-off. Adding tokens, tools, or weight-level circuitry increases parameter count and training complexity and raises orchestration and verification burden. At the same time, it can markedly reduce wall-clock latency, exploration cost, and the memory pressure of long reasoning traces. For modern LLMs, the balance differs from classical CPUs: scaling parameter count is comparatively straightforward and efficient, while long sequential chains of thought are expensive, hard to parallelize, and brittle with respect to context length and recall.

These constraints suggest a natural design pressure toward *maximalistic models of computation*: computational engines equipped with rich tool libraries, learned subcircuits for frequent subproblems, and token types representing complex operations. Concretely, this includes (i) a library of skills—APIs, theorem banks, solvers—with learnable dispatch, (ii) token or type extensions that encode compound operators, and (iii) fine-tuned subnets that implement high-value routines (e.g., parsing, algebraic transforms, approximate simulation). Training signals should reward short-horizon solutions and penalize unnecessary long-form reasoning when a reliable jump exists.

Maximalistic models of computation stand in contrast with *minimalistic* ones, such as Turing machines, which are designed to operate with the smallest possible instruction set on the smallest possible dictionary through a computer with the smallest number of components. Such simplicity makes sense if the goal is analysis by humans. However, beyond interpretability, this pressure towards simplicity does not reflect the structure of the world that an AI agent must interact with. Such a world instead engenders pressure towards speed, which can be optimized by increased complexity.

Maximalism is not mere memorization. Automatized routines and abstracted lemmas are structured compressions of procedures, not rote patterns. They improve reliability and latency on recurring structures (FFT, parsing, algebraic simplifications), while remaining fallible on rare, non-stationary, or adversarial cases where general reasoning must reassert itself. Selecting which operations deserve “instruction status”— balancing parameter growth against step reduction—and validating the safety of state jumps is a key problem for reasoning LLMs.

As minimizing reasoning-trace length becomes the dominant objective, we should expect models to shift toward maximalistic computation, mirroring the biological transition from System-2 deliberation to System-1 fluency. The path to greater speed is not only better search, but better *steps*.

## Appendix B. System–Theoretic Notation

Since LLMs are stochastic dynamical systems, in this section, we write the definition of proper time in notation more familiar to those with system theory background, where a discrete-time, discrete-state stochastic dynamical model is expressed in the form

(A1) $$\left\{\begin{array}{c} x_{t+1}=f\left(x_{t}\right)+g\left(x_{t},u_{t}\right)+v_{t}\\ y_{t}=h\left(x_{t}\right)+n_{t} \end{array}\right.$$

where *x* is the state, *f* the drift vector field, *g* the input map, *v* the driving noise, *h* the output map, and *n* the measurement noise. The following table maps these terms to the notation in this paper.

**Table A1:** Summary of notation.

	Automata	System Theory
state	*s*	*x*
state transition	ν(·|·)	p(·|·)
state trajectory	$h=(s_{1},\ldots ,s_{n})$	$x_{1:t}$
output map	a=dec(s)	y=h(x)
input map	s=enc(u)	g(u)
output	$h_{n}\Rightarrow a$	$\Phi \left(x_{0},u_{1:t}\right)=y_{1:t}$

Let $x_{1:t}$ be a state trajectory starting from $x_{0}$ driven by input $u_{1:t}$, which may include a stochastic component. The transition probability is $p(x_{i+1}|x_{i},u_{i})$ for all i=1,…,t. We denote with $x_{t}=\Phi \left(x_{0},u_{1:t}\right)$ the *flow* of the system from $x_{0}$ to $x_{t}$ under input $u_{1:t}$, and $P\left(x_{t}|x_{0},u_{1:t}\right)=\prod_{i=1}^{t}p\left(x_{i+1}|x_{i},u_{i}\right)$ is the corresponding probability. A target state $x_{t}$ is *reachable* from $x_{0}$ if there exits at least one such input. We denote by $R_{t}\left(x_{0}\right)=\left\{x_{t}\;s.\;t.\;\exists \;u_{1:t}\;|\;x_{t}=\Phi \left(x_{0},u_{1:t}\right)\right\}$ the set of states reachable in *t* steps, and $R\left(x_{0}\right)=\left\{R_{t}\left(x_{0}\right),\;t\in N\right\}$ is the set of states reachable from $x_{0}$. Finally, the reachable set $R=\{R\left(x_{0}\right),\;|\;x_{0}\in X\}$ is the set reachable from some initial state. Proper time is then defined as

$$\tau \left(x_{a},x_{b}\right)=\min_{t\;|x_{b}\in R_{t}\left(x_{a}\right)}\frac{t}{P(x_{b}|x_{a},u)}$$

where *u* is any *t*-long input such that $x_{b}=\Phi \left(x_{a},u\right)$. Alternatively, we can write

$$\tau \left(x_{a},x_{b}\right)=\min_{u\;|\;x_{b}=\Phi \left(x_{a},u\right)}\frac{\left|u\right|}{P(x_{b}|x_{a},u)}$$

The reachable set can be written as the indicator function of the verifier $R\left(x\right)=\chi_{\left\{y\;|\;f(x,y)=1\right\}}$.
